# Testing psychosocial work adversities as a necessary condition for work-related emotional exhaustion in young workers: a cross-sectional necessary condition analysis on a national general working population-based survey

**DOI:** 10.1136/bmjopen-2024-094485

**Published:** 2025-11-12

**Authors:** Roosmarijn M.C. Schelvis, Malte van Veen, Sietske J. Tamminga, Karen M. Oude Hengel, Karen Nieuwenhuijsen, Cécile R.L. Boot, Jan Dul

**Affiliations:** 1 Public and Occupational Health, Amsterdam UMC Location AMC, Amsterdam, Netherlands; 2 TNO/Amsterdam UMC/APH, Body at Work, Research Center on Work, Health and Technology, Amsterdam, Netherlands; 3 Amsterdam Public Health, Societal Participation & Health, Amsterdam, Netherlands; 4 Unit Healthy Living & Work, TNO Location Leiden Sylviusweg, Leiden, Netherlands; 5 Public and Occupational Health, Amsterdam UMC Location VUmc, Amsterdam, Netherlands; 6 Rotterdam School of Management, Erasmus University Rotterdam, Rotterdam, Netherlands

**Keywords:** MENTAL HEALTH, Occupational Stress, Cross-Sectional Studies

## Abstract

**Abstract:**

**Objectives:**

Being exposed to adverse psychosocial working conditions contributes to poor mental health in young workers. This study explores whether psychosocial work adversities are a necessary condition for work-related emotional exhaustion in young workers.

**Design:**

Data from the ‘Netherlands Working Condition Survey 2021’ was used. By applying a novel method called Necessary Condition Analysis, we tested two psychosocial work adversities as necessary conditions for high work-related emotional exhaustion in young workers: (1) a composite score of high job demands and low job resources and (2) a composite score of high job demands. Additionally, we tested whether the threshold for job demands as a necessary condition for high work-related emotional exhaustion differed for young workers with low versus high resources.

**Setting:**

Secondary data analysis on a national working population-based survey.

**Participants:**

The sample included 5791 young workers in the Netherlands (aged <30 years; 56.8% female).

**Primary outcome measure:**

Work-related emotional exhaustion.

**Results:**

A high level of the composite on job demands and job resources is necessary for a high level of work-related emotional exhaustion in young workers (effect size=0.11, p<0.001), and the same applies to the composite score of high job demands alone (effect size=0.10, p<0.001). The necessity threshold for job demands, which guarantees the absence of a particularly high level of work-related emotional exhaustion, was higher for the group of young workers with high job resources compared with young workers with low job resources.

**Conclusions:**

Both psychosocial work adversities were necessary conditions for high work-related emotional exhaustion in young workers. The necessity threshold for job demands was higher for young workers with high job resources, compared with the group with low resources. This indicates that removing psychosocial work adversities and ensuring the presence of job resources might contribute to the prevention of high work-related emotional exhaustion in young workers.

**STRENGTHS AND LIMITATIONS OF THIS STUDY**

- A methodological strength is that the outcomes of our Necessary Condition Analyses apply to virtually all cases of young workers.
- Another methodological strength of this study is the replication of the analysis in seven additional waves of the same study.
- Due to the cross-sectional nature of this study, reciprocal or reversed causality cannot be ruled out completely.
- The credibility of the necessity relationship found would increase with a time-lagged, longitudinal or experimental study design.

## Introduction

 Being exposed to adverse psychosocial working conditions contributes to adverse mental health in employees,^1 2^ and it is argued that this is especially true for young workers aged ≤30 years.^3^ However, exposure to adverse psychosocial working conditions has been studied mainly from the perspective of probabilistic causality, which seems inherent to the most commonly applied statistical models in the fields of occupational health and epidemiology. The matter has since long been the subject of debate, with strong advocates of the probabilistic view.^4 5^ In the dominant probabilistic causal perspective, the cause (psychosocial working conditions) is assumed to have an effect on the outcome (mental health) *on average*. Although knowledge about average effects is important, it implies that a large part of the population will not experience the positive effects or might even experience a negative effect. The large number of studies that applied this reasoning has brought us a long list of possible determinants that could all likely, on average, positively or negatively contribute to adverse mental health.

Recently, a different perspective on studying the relationship between two variables has entered a variety of disciplines in the social, medical and technical sciences (eg,^6^,^8^ respectively): necessity logic and accompanying Necessary Condition Analysis (NCA).^9^ Necessity logic takes a deterministic necessity perspective (if not X then not Y) in contrast to the common probabilistic sufficiency perspective (if X then probably Y). Necessary conditions are ‘critical’ factors that can be observed in every single case in which an outcome is present. Without the factor, the outcome does not occur and another factor cannot compensate for the absence of the necessary factor. NCA allows for ‘in degree statements’: there must be a certain level of X to have a certain level of Y. In this article, we introduce this perspective to occupational health research by examining the relationship between adverse psychosocial working conditions and adverse mental health in young workers from a necessity logic point of view.

**Figure 1:**
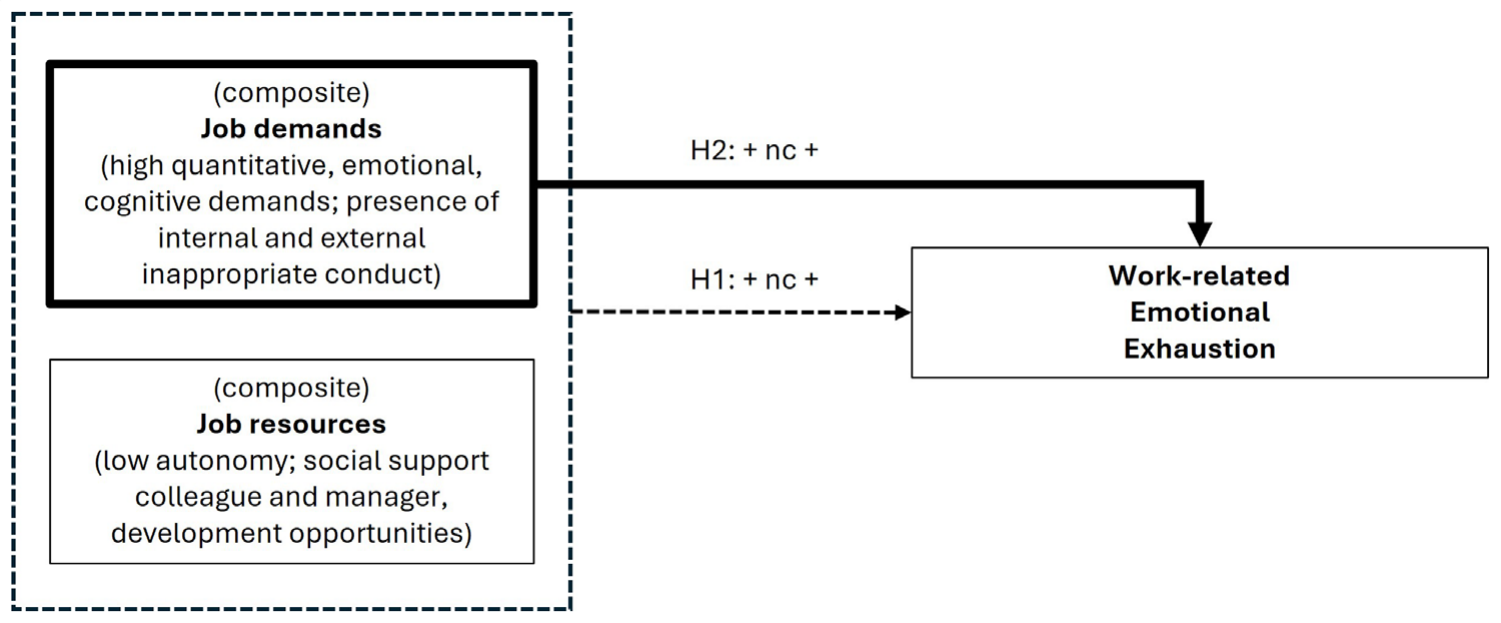
Necessity hypotheses within the Job Demands Resources framework.

Our research is inspired by the Job Demands Resources (JDR) model which distinguishes between work characteristics as either job demands or job resources.^10^ According to the JDR model, job resources can fulfil psychological needs and can therefore buffer the negative impact of job demands on work-related mental health.^11^ As no consensus is at hand for the operationalisation of adverse work-related mental health, we have chosen work-related ‘emotional exhaustion’ as operationalisation.^12^ Since earlier research suggests that there is not one toxic factor explaining mental health outcomes,^2^ we consider emotional exhaustion to be an equifinal phenomenon (ie, a different combination of job demands and job resources per individual worker can lead to the same degree of emotional exhaustion). Against this background, we hypothesise that (figure 1):

H1. A high level on the *composite score of job demands and job resources* is necessary for a high level of *work-related emotional exhaustion* in young workers;

H2. A high level of *job demands* is necessary for a high level of *work-related emotional exhaustion* in young workers.

We additionally test whether the hypothesised necessity differs for young workers with *high versus low job resources*.

## Methods

### Study population and ethics approval

Data from the 2021 wave of the Netherlands Working Condition Survey (NWCS) were used, which is an annual cross-sectional survey to monitor the health and working conditions of Dutch workers aged 15–74 (n=49 659). The original methodological report on this survey in Dutch can be found elsewhere,^12^ an English translation of the ‘data collection and processing’ chapter is available as online supplemental file 1. According to the Netherlands Organisation for Applied Scientific Research TNO’s Internal Review Board, the NWCS was not subject to the requirements of the Dutch Medical Research Involving Human Subjects Act and the study was approved (approval number 2018–066).

For the current study, young workers aged 18 until and including 29 (n=9191) were eligible and they needed to work at least 16 hours in a paid job (n=6115). They further had to have data for emotional exhaustion and psychosocial work variables resulting in a final sample of 5791 young workers.

### Outcome variable: work-related emotional exhaustion

Work-related emotional exhaustion was measured with the emotional exhaustion scale of the validated Utrecht Burnout Scale^13^ which is an adjusted Dutch version of the Maslach Burnout Inventory-General Survey (MBI-GS).^14^ The subscale consisted of five items (eg, “I feel emotionally exhausted by my work”) and the seven-point response scale ranged from (1) “never” to (7) “every day” (online supplemental information, table 1). The work-related emotional exhaustion score was calculated as the mean score of the items, with higher scores indicating more emotional exhaustion. Internal consistency of the scale in this sample was good (Cronbach’s alpha=0.89; online supplemental information, table 1).

### Exposure variables

#### Job demands and job resources variables

The variables were selected based on discussions within the project team and their availability in the dataset (online supplemental information, table 1 for item origin, all items of the scales used, answer categories for all variables). Cronbach’s alphas for all scales were calculated to establish the internal consistency, with acceptable to good results (lowest Cronbach’s alpha=0.75; highest Cronbach’s alpha=0.85; online supplemental information, table 1). Five job demand variables were included: quantitative demands (three items), emotional demands (three items), cognitive demands (three items), internal (four items) and external inappropriate conduct (four items). Four job resource variables were included: autonomy (six items), colleague support (two items), manager support (two items) and development opportunities (one item).

#### Constructing composite scores for hypothesis testing

Three composite scores were calculated for psychosocial work adversities: job demands, job resources and a combination of high job demands and low job resources. First, all variables were coded in the same direction with a higher score reflecting more adversity. Next, all variables were rescaled so that the minimum score was 0 (=no adversity) and the maximum score was 1 (highest adversity), preserving the individual distribution per variable. For the demands ‘internal and external inappropriate conduct’, every experience was considered as adverse, so all ‘Yes’-answer categories (2, 3 and 4) received a score of 1. Subsequently, the mean of all variables was calculated per composite, meaning that scoring the maximum level on all factors would result in a composite score of 1 and scoring the minimum score on all factors would result in a composite score of 0. The composite score on job demands included the five job demand variables, the composite score on job resources included the four job resource variables, the composite score on high demands and low resources included all nine variables. The composites are treated as continuous variables, except in the sensitivity analysis accompanying hypothesis two for which job resources were dichotomised into low and high job resources based on a median split.

### Data analysis

We applied NCA^9^ to hypotheses H1 and H2 on psychosocial work adversity as necessity for work-related emotional exhaustion first for the whole sample of young workers and then for young workers with high versus low job resources separately. We not only test the *composite* as necessary conditions (eg, job demands), but also the *scales* (eg, cognitive demands), and also all individual *items* within the scales (eg, “does your work require intense thinking?”). This is done in order to assess if one of the underlying scales or items is driving the necessity association.

NCA is a multiple bivariate analysis that identifies empty spaces in scatterplots that are compatible with the hypothesis (in the current case upper left corner) by drawing a ceiling line on top of the data, which acts as the border between the empty and the full area. Visual inspection of scatter plots was used to identify a potential necessary condition and to select the ceiling line. We selected the stepwise Ceiling Envelopment - Free Disposal Hull (CE-FDH) ceiling line because the border was not linear.^15^ Effect sizes were calculated as is suggested for NCA by dividing the ceiling zone, which is the size of the empty space, by the empirical scope, which is the area of the scatter plot bounded by the minimum and maximum values of the variables. As a rule of thumb, effect sizes can be small (0<d<0.1), medium (0.1≤d<0.3), large (0.3≤d<0.5) or very large (d≥0.5).^16^ We considered an effect size of d=0.05 as relevant given the practical importance of our outcome. Using a permutation test,^16^ a p value is estimated, which is the probability that the effect size is compatible with effect sizes produced by unrelated variables. Since NCA is essentially not a statistical but a mathematical method, no confidence interval (CI) for NCA exists. Uncertainties are not expressed in probabilities but in ‘cardinalities’ (eg, number of cases that do not comply: exceptions). Necessary conditions operate in isolation from the rest of the causal structure, such that control variables and confounders are not relevant for the analysis. Bottleneck tables were used to quantify which level of the composite psychosocial work adversities is necessary for a particular level of work-related emotional exhaustion.

We tested the robustness of findings with seven earlier waves of the same survey (2014 till 2020), thereby including 49 454 young workers in total. All analyses were conducted using the NCA package version 3.3.3 in R 4.0.2 in RStudio Version 1.3.959.

### Outlier analysis

Outlier identification is important in a large dataset as in the current study (n=5791). The effect size is susceptible to single data points that determine the ceiling line or the scope area. We used the NCA R-package for outlier detection, and we considered cases which affected the effect size by 30% or more as anomalies. We followed a pragmatic necessity logic and thus allowed for the exclusion of a number of cases in further reporting, to come to a statement relevant for virtually all young workers. No outliers were identified for psychosocial work adversities, while one outlier was identified for job demands as a necessary condition was identified. All effect sizes and p values are reported after exclusion of the identified outlier.

## Results

### Participant characteristics

The sample consisted of slightly more females (57%) than males and the mean age was 25 years (table 1). About half of the sample had higher secondary educational training (53%) and the large majority had a permanent employment contract with fixed working hours (70%). The position in the household differed between the young workers: almost 39% lived with (one or more) parents or caregivers, 19% lived on their own and 40% lived with a partner, of which a minority also cared for children (table 1).

**Table 1:** Characteristics of study population (n=5791).

Characteristics	Count (frequency)	Mean (SD)
Gender		
Female	2503 (56.8%)	
Male	3288 (43.2%)	
Age (min 18.0; max 29.0)		24.9 (3.07)
Working hours per week (min 17.0; max 95.0)		34.3 (6.91)
Educational level*		
Elementary†	488 (8.4%)	
Vocational‡	2225 (38.4%)	
Academic§	3043 (52.5%)	
Type of employment		
Permanent employment, fixed hours	4057 (70.1%)	
Prospect of permanent employment, fixed hours	512 (8.8%)	
Temporary employment, fixed hours	215 (3.7%)	
Temporary or on call employee	420 (7.9%)	
Permanent or temporary employment without fixed hours	351 (6.1%)	
Household composition		
Child living with parent(s) or caregiver(s)	2233 (38.6%)	
Single person	1114 (19.2%)	
Partner in (un)married couple without children	1942 (33.5%)	
Partner in (un)married couple with children	387 (6.7%)	
Parent in single-parent household or other household	115 (2%)	

* Missings in educational level n=44 (0.7%).

† Elementary education represents a maximum of 1 year of completed vocational education.

‡ Vocational education represents more than 1 year of completed vocational education without completed academic education.

§ Academic education represents a bachelor’s degree from a university or university of applied sciences.

### H1: composite of high demands and low resources is necessary for work-related emotional exhaustion

**Figure 2:**
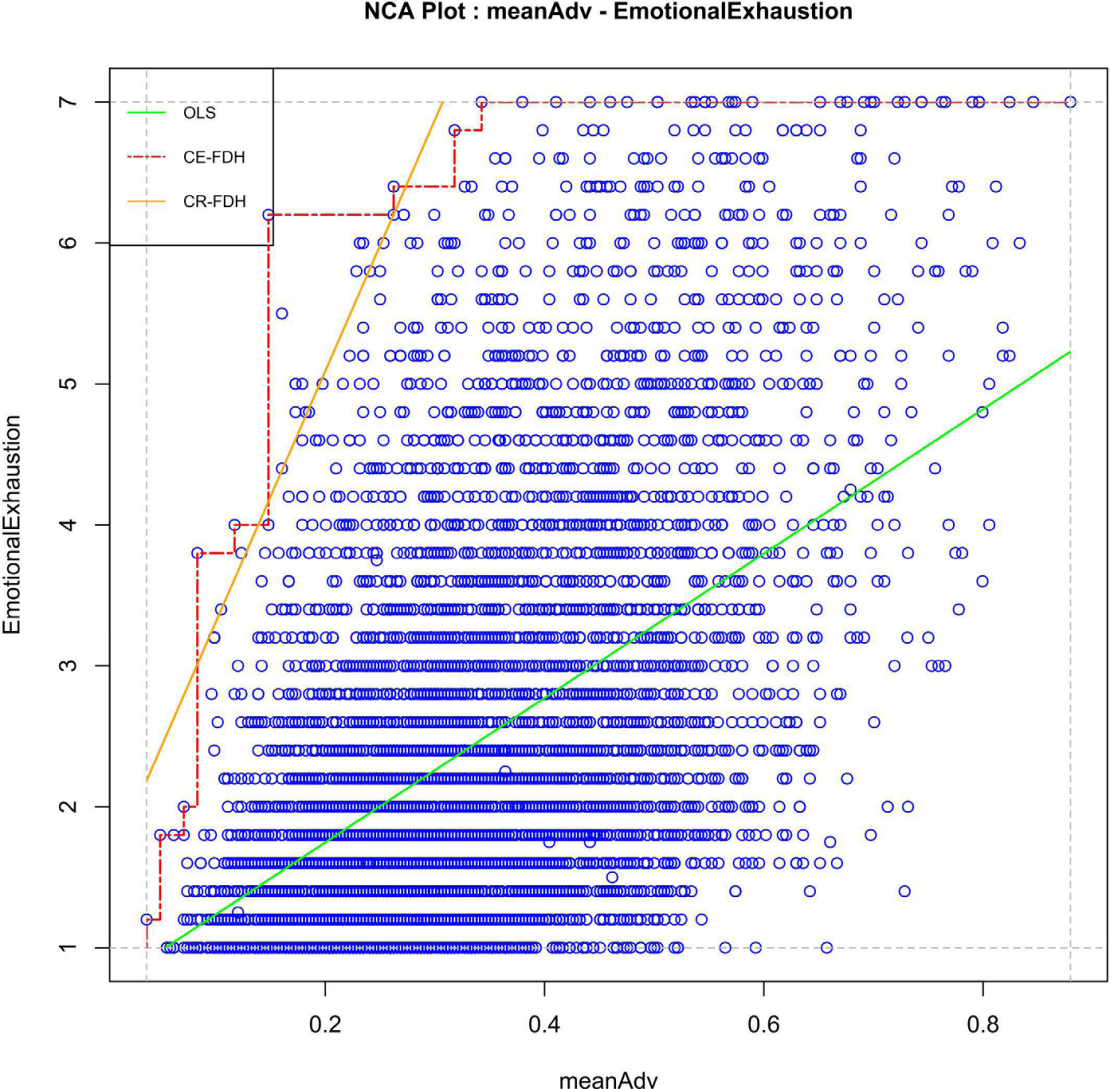
Scatterplot for composite high job demands and low job resources (**x**) and work-related emotional exhaustion (**y**) without outliers. Note: CE-FDH is the stepwise Ceiling Envelopment - Free Disposal Hull line (red, dashed line); CR-FDH is the Ceiling Regression - Free Disposal Hull line (yellow, upper solid line); OLS is the Ordinary Least Squares regression line (green, lower solid line).

A high level of the composite on job demands and job resources is necessary for a high level of work-related emotional exhaustion in young workers (empty upper left corner in figure 2; effect size=0.11, p<0.001). This means that a high level of psychosocial work adversities must be present in a young worker in order to also have a high level of work-related emotional exhaustion, regardless of other factors, supporting our hypothesis. The bottleneck table shows that for experiencing a work-related emotional exhaustion level of 4 (ie, ‘several times per month’), a young workers’ score on the demands and resources composite is necessarily 0.12, whereas a composite score of 0.15 is necessary for an exhaustion score of 6 (ie, ‘several times a week’) and 0.34 for an exhaustion score of 7 (ie, ‘every day’; table 2).

**Table 2:** Bottleneck table based on Ceiling Envelopment - Free Disposal Hull (CE-FDH) line, after outlier exclusion.

Work-related emotional exhaustion	Composite job demands and job resources	Composite job demands in low job resource group	Composite job demands in high job resource group
Never (1)	NN*	NN*	NN*
Several times a year (2)	0.071	0.022	0.044
Monthly (3)	0.083	0.022	0.067
Several times a month (4)	0.117	0.089	0.111
Every week (5)	0.148	0.089	0.156
Several times a week (6)	0.148	0.111	0.200
Every day (7)	0.343	0.378	0.400

Note: a complete bottleneck table with substeps of 0.2 is presented in online supplemental information, table 2.

* NN: not necessary, meaning that for a level of 1 (never) on work-related emotional exhaustion (Y) no minimum level of psychosocial work adversity (X) is required.

On further analysis, none of the job demand scales or job resource scales in the composite were necessary conditions (all effect sizes≤0.01) nor were items within these scales necessary conditions (all effect sizes≤0.02). This indicates that none of the scales or items alone are responsible for the necessity we found in the composite.

### H2: composite job demands is necessary for work-related emotional exhaustion and the level of job resources matters

**Figure 3:**
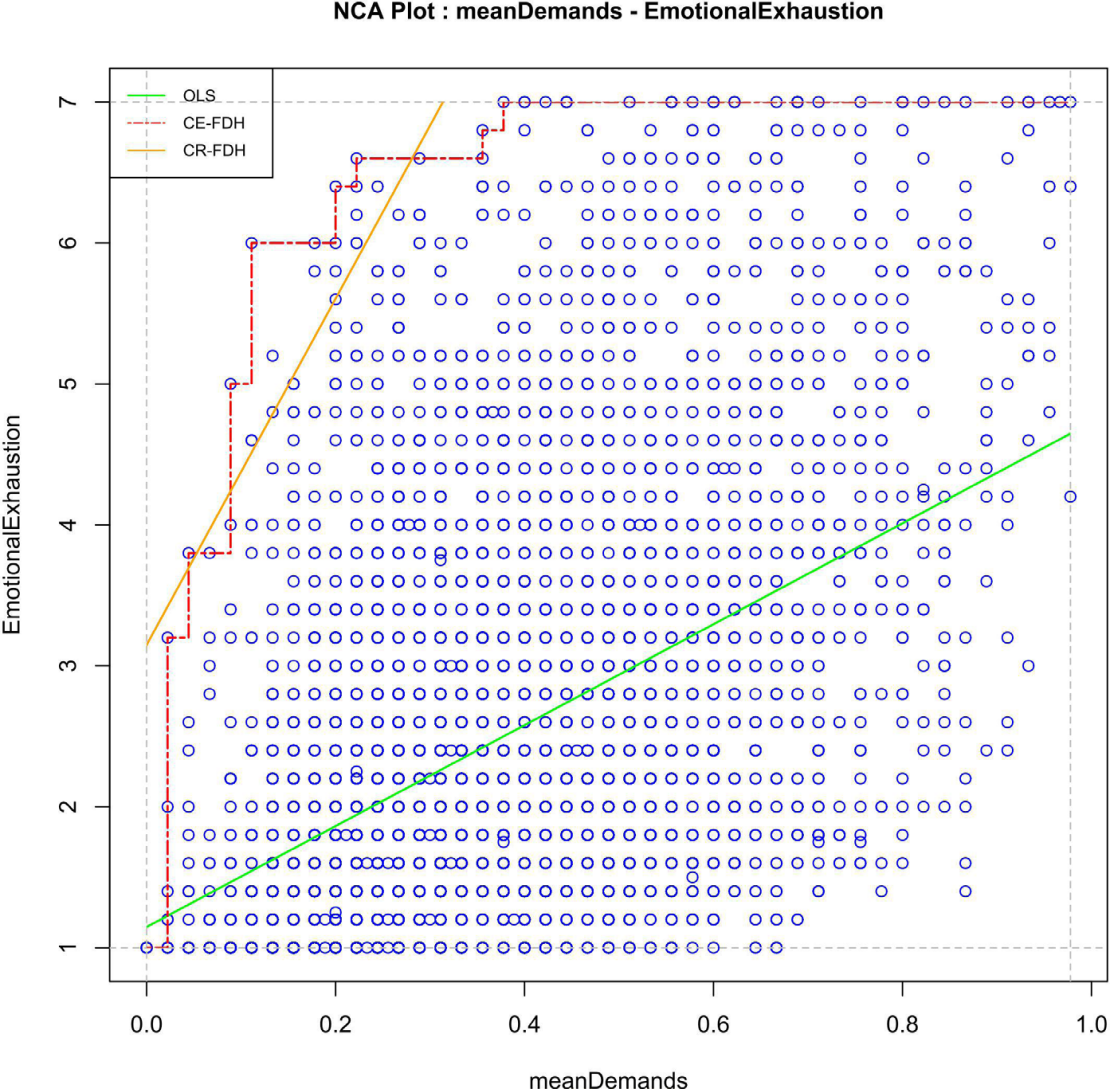
Scatterplot for composite job demands (**x**) and work-related emotional exhaustion (**y**) without outliers. Note: CE-FDH is the stepwise Ceiling Envelopment - Free Disposal Hull line (red, dashed line); CR-FDH is the Ceiling Regression - Free Disposal Hull line (yellow, upper solid line); OLS is the Ordinary Least Squares regression line (green, lower solid line).

In line with our second hypothesis, we found that a high level of the composite job demands is necessary for a high level of work-related emotional exhaustion in young workers (empty upper left corner in figure 3; effect size=0.10, p<0.001), regardless of the level of job resources (figure 3). In the group of young workers with low resources, a high level of job demands was necessary for a high level of emotional exhaustion (effect size=0.10, p<0.001). Whereas in the group with high resources, a higher level of job demands was necessary for a high level of emotional exhaustion, as indicated by a lower ceiling line (effect size=0.15, p<0.001). More specifically, the bottleneck table demonstrates that for reaching an emotional exhaustion level of 4 (ie, ‘several times a month’), the high resource group needs a composite job demands score of 0.11, whereas a score of 0.09 is already necessary for reaching that same level in the group with low resources (table 2). In conclusion, the necessity threshold for job demands differed for young workers with low versus high resources.

### Robustness of findings

The findings for hypothesis 1 were replicated in the 2014–2020 waves of the NWCS. All effect sizes were at least 0.08, with p<0.001 (online supplemental information, table 1). The same holds for hypothesis 2, the findings could be replicated as well in the 2014–2020 waves of the NWCS. All effect sizes for the low resource group were at least 0.05 with p<0.001, and all effect sizes for the high resource group were at least 0.10 with p<0.01 for all but 1 year (online supplemental information, table 3). Lastly, we performed an additional sensitivity analysis on the 2021 sample, using quartiles as a cut-off instead of median split for creating groups of young workers with lower versus higher levels of job resources. We could replicate our finding and found in addition that the higher the job resource score, the larger the effect size (online supplemental information, figure 1; q1=0.10; q2=0.12; q3=0.15; q4=0.20; all p<0.001).

## Discussion

Using necessity logic and the NCA,^9^ we hypothesised that psychosocial work adversities are necessary for high work-related emotional exhaustion in young workers. First, we tested a composite score of high job demands and low job resources (psychosocial work adversities), and second a composite score of high job demands as necessities. Both composites were necessary conditions for work-related emotional exhaustion. Additionally, we tested whether the necessity threshold for job demands differed for young workers with low versus high resources. For young workers with high job resources, the amount of job demands could be higher before they became necessary for a high level of work-related emotional exhaustion, compared with young workers with low job resources.

### Reflection on findings

Probability-based research can only capture effects that are averaged over (often) heterogeneous populations. Necessity logic provides a different lens for understanding how psychosocial work exposures affect work-related emotional exhaustion. In NCA, the results apply to virtually all participants and enable the identification of necessary conditions that must be present for enabling a desired outcome (eg, good mental health) or must be absent for blocking an undesired outcome (eg, mental health problems). The necessity of psychosocial work adversities for work-related emotional exhaustion suggests that removing high levels of psychosocial work adversities will avoid high levels of emotional exhaustion in nearly all cases. Moreover, the levels of x and y are shown in the bottleneck table, but the interpretation of what level of exposure and outcome is (too) high needs discussion among scholars and practitioners as well as between managers and employees within organisations: is it acceptable to experience work-related emotional exhaustion several times a year, monthly or every week, and under what conditions? An interdisciplinary group of authors from science, practice and policy described in a recent discussion paper^17^ the well-known difficulty of determining occupational exposure limits for psychosocial risk factors in the workplace. The authors proposed a stepwise process of joint action with all relevant stakeholders (eg, scientists, practitioners, policy-makers), ranging from *conceptual agreement* to experimental and epidemiological evidence, leading to *consensus* and ultimately the establishment of *occupational exposure limits*. Our results, as well as future NCA analyses for other subgroups or even the entire working population, could inform this process and complement the proposed NOAEL (no-observed-adverse-effect level) and LOAEL (lowest-observed-adverse-effect level) methods, as NCA results apply to (virtually) all cases.

Our study is among the first to study psychosocial exposures and a mental health outcome in the workplace from a necessity perspective. One recent study by Manchiraju and colleagues^18^ on entrepreneurial role stress and burnout demonstrated the necessity of a role stress composite score (ie, role conflict, role ambiguity and role overload) for the presence of burnout in entrepreneurs. Our study differs from this study in the domain (young workers in organisations vs self-reported entrepreneurs), the theoretical mental health concept (JDR model^10^ vs the ‘tri-component conceptualisation of role stress’^19^) and the measurements of the outcome (Utrecht Burnout Scale^13^ vs the burnout scale by Malach-Pines^20^).

### Methodological considerations

A limitation of our study is that it is based on cross-sectional data. The occurrence of reverse and reciprocal causality between mental health outcomes and psychosocial adversities is still a matter of debate,^15 21^ and work-related emotional exhaustion has also been shown to change perceptions of job demands and resources.^22^ Consequently, we cannot rule out that high emotional exhaustion itself contributes to higher (experienced) psychosocial work adversities. In general, necessary conditions that are identified in a cross-sectional study may be causally interpreted only if there is theoretical support for it and if reverse causality is considered implausible. The credibility of a causal necessity relationship increases with a time-lagged, longitudinal or experimental study design.^23^ Further research of this type is needed to rule out reciprocal or reversed causality.

We developed composite scales for testing psychosocial work adversities. Even though information from individual items might be lost and measurement error cannot be accounted for, this approach makes sense theoretically because there is not one toxic factor for work-related emotional exhaustion. Empirically, this is confirmed by the finding that none of the demand or resource scales, nor any of the items within these scales, were necessary conditions, which gives us confidence in the appropriateness of our procedure to create composite scales. As a test for robustness, we replicated the findings for both hypotheses in seven earlier waves of the same survey. For the second hypothesis, an additional sensitivity analysis on the 2021 sample using quartiles of job resources instead of high versus low job resources was performed, which showed that the higher the job resources, the higher the effect size, indicating a stronger necessary association. All results were statistically significant and effect sizes were small to medium (online supplemental information, tables 3,4). Despite the observed necessity to infer causal necessity beyond theoretical reasoning, a longitudinal dataset with multiple datapoints per participant would be helpful.

### Implications for research and practice

Not all young workers with high psychosocial work adversities report high work-related emotional exhaustion. Future research could look into other contributing factors that determine high emotional exhaustion when high work adversities are present (eg, coping style, personal characteristics, a history of poor mental health, or organisational factors).

Our study showed that work-related emotional exhaustion was observed in young workers with high job demands. However, in workers with a high level of resources, the threshold for job demands being a necessary condition for exhaustion was higher. This is in line with the ‘buffering hypothesis’ of the JDR model^24^ and future research could clarify the mechanisms behind the shift in the NCA ceiling line that we observed.

We drafted our hypotheses in line with necessity logic’s enabling formulation (one must have (level of) X to have (level of) Y). The logical equivalent of these hypotheses is the constraining formulation (the absence of X is sufficient for the absence of Y). Lowering the level of adversities guarantees a reduction of the maximum level of emotional exhaustion. Comparable to the ‘safe dose’ of exposure to potentially harmful agents, our results might inform specific targets or maximum levels of adversities in work organisations.

### Concluding remarks

In our experience, necessity logic and NCA are an enrichment to occupational health research. We conclude that without psychosocial work adversities, high work-related emotional exhaustion is absent in young workers. This might indicate a necessity role of psychosocial work adversities for young workers’ mental health. We illustrated how NCA logic and method requires different theorising before the start of the study than what we are used to in our field.

## Supplementary material

10.1136/bmjopen-2024-094485 online supplemental file 1

10.1136/bmjopen-2024-094485 online supplemental file 2

## Data Availability

No data are available.

## References

[1] Butterworth P, Leach LS, Strazdins L (2011). The psychosocial quality of work determines whether employment has benefits for mental health: results from a longitudinal national household panel survey. Occup Environ Med.

[2] Harvey SB, Modini M, Joyce S (2017). Can work make you mentally ill? A systematic meta-review of work-related risk factors for common mental health problems. Occup Environ Med.

[3] Shields M, Dimov S, Kavanagh A (2021). How do employment conditions and psychosocial workplace exposures impact the mental health of young workers? A systematic review. Soc Psychiatry Psychiatr Epidemiol.

[4] Parascandola M, Weed DL (2001). Causation in epidemiology. J Epidemiol Community Health.

[5] Vreese L (2009). Epidemiology, Medicine. Health Care and Philosophy.

[6] Karwowski M, Dul J, Gralewski J (2016). Is creativity without intelligence possible? A Necessary Condition Analysis. Intelligence.

[7] Luther L, Bonfils K, Firmin R (2017). M34. Metacognition is Necessary for the Emergence of Motivation in Schizophrenia: A Necessary Condition Analysis. Schizophr Bull.

[8] Kwon SP (2021). Examination of design parameters affecting Nb _3_ Sn CICC current sharing temperature using necessary condition analysis. Supercond Sci Technol.

[9] Dul J (2015). Necessary Condition Analysis (NCA): Logic and Methodology of “Necessary but Not Sufficient” Causality. Organ Res Methods.

[10] Demerouti E, Bakker AB, Nachreiner F (2001). The job demands-resources model of burnout. J Appl Psychol.

[11] Bakker AB, de Vries JD (2021). Job Demands–Resources theory and self-regulation: new explanations and remedies for job burnout. *Anxiety, Stress, & Coping*.

[12] Dam L, Mars G, Knops J (2022). Nationale Enquête Arbeidsomstandigheden 2021. Methodologie.

[13] Schaufeli WB, Dierendonck D (2000). UBOS Utrechtse Burnout Schaal: Handleiding.

[14] Maslach C, Jackson S, Leiter M (1986). Maslach Burnout Inventory Manual Consulting Psychologists Press.

[15] Shahidi FV, Smith PM, Oudyk J (2022). Longitudinal Reciprocal Relationships Between the Psychosocial Work Environment and Burnout. J Occup Environ Med.

[16] Dul J, van der Laan E, Kuik R (2020). A Statistical Significance Test for Necessary Condition Analysis. Organ Res Methods.

[17] Pauli R, Lang J, Müller A (2025). Requirements for occupational exposure limits in psychosocial risk assessment: What we know, what we don’t know and what we can learn from other disciplines. Scand J Work Environ Health.

[18] Manchiraju S, Akbari M, Seydavi M (2024). Is entrepreneurial role stress a necessary condition for burnout? A necessary condition analysis. Curr Psychol.

[19] Buttner EH (1992). Entrepreneurial stress: is it hazardous to your health. Journal of Managerial Issues.

[20] Malach-Pines A (2005). The Burnout Measure, Short Version. Int J Stress Manag.

[21] ter Doest L, De Jonge J (2006). Testing causal models of job characteristics and employee well‐being: A replication study using cross‐lagged structural equation modelling. J Occupat & Organ Psyc.

[22] Guthier C, Dormann C, Voelkle MC (2020). Reciprocal effects between job stressors and burnout: A continuous time meta-analysis of longitudinal studies. Psychol Bull.

[23] Dul J (2022). Advances in necessary condition analysis. downloaded on jan 2021.

[24] Bakker AB, Demerouti E, Euwema MC (2005). Job resources buffer the impact of job demands on burnout. J Occup Health Psychol.

