# Supplementary material for: Testing psychosocial work adversities as a necessary condition for work-related emotional exhaustion in young workers: a cross-sectional necessary condition analysis on a national general working population-based survey
Source: BMJ Open. 2025 Nov 12;15(11):e094485. doi: 10.1136/bmjopen-2024-094485 (PMC12612735; doi:10.1136/bmjopen-2024-094485)
Supplement: online supplemental file 1 [file bmjopen-15-11-s001.pdf]

Subscribe to DeepL Pro to translate larger documents.
Visit www.DeepL.com/pro for more information.

3 DATA COLLECTION AND PROCESSING

3.1 PRELIMINARY DESIGN

Individuals from the sample can participate in the survey via the internet (computer-assisted web interviewing, CAWI). Statistics Netherlands (CBS) carries out the fieldwork using the following strategy:

- Sample individuals receive a letter containing 1) a request to participate in the survey via the internet and 2) the corresponding login details. The letter is accompanied by a brochure specifically compiled for the NEA.
- Two weeks later, a first reminder letter is sent to sample subjects, again requesting them to respond via the internet.
- Five weeks after the letter, a second reminder letter is sent.
- Seven weeks after the initial letter, a third reminder letter is sent. This letter also states the deadline for completing the online questionnaire.

All reminder letters are only sent to sample subjects who did not respond at the time of selection and who did not indicate via the Contact Center Inbound (CCI) that they did not want to or could not participate in the survey.

Part of the approach design is a conditional incentive strategy. The initial letter and the three reminder letters state that respondents have a chance to receive a conditional reward worth €250. in the form of gift cards. In addition, CBS has a policy of asking parents/guardians for permission for persons aged 12 to 15 to participate in the survey. This is no longer done for persons aged 16 and older. Sample persons aged 15 therefore receive a version of the invitation and reminder letters that is addressed to their parents or guardians.

The letters mentioning the possibility of winning €250 state the following:

- If a sample subject responds, he or she will know immediately afterwards whether the reward has been won.
- In the questionnaire, a potential winner can indicate that they do not wish to claim the reward, the so-called opt-out option.

The letters are signed only by CBS, and only the CBS logo is used in communications with respondents. Respondents can contact CBS with questions by email, telephone (on working days from 9:00 a.m. to 5:00 p.m.), or via the internet. The contact details are stated in the letter. Persons in the sample are informed that this is a joint study by TNO, SZW, and CBS and that CBS will supplement the collected data with data already available at CBS. See section 3.7 for more information about the protection of personal data.

3.1.1 Expected response

The CBS and TNO aim to achieve a total response rate of 30.5%. The preliminary design of the NEA 2021 is largely similar to that of the NEA 2020, as are the preconditions for the sample design (see section 3.2.3). In 2020, this resulted in a higher response rate of 36.7%. The sample design for the NEA 2021 is based on the response rate achieved for the NEA 2020, or more precisely, on the usable responses achieved. This is because some of the responses ultimately prove to be unusable for publication purposes. There may be dropouts due to framework errors or insufficient completion of a completed questionnaire. Based on the approach and sample design (see section 3.2.3) and the actual response rate for the NEA 2020, it is expected that 57,953 usable responses will be achieved.

3.1.2 Fieldwork period

The sample for the NEA will be sent out in six batches. The letters for the first batch will be sent out on September 21, 2021, and those for the sixth batch on October 26, 2021. The letters and reminders will be sent on Tuesday by non-time-critical mail, which means that respondents will receive the letters on Thursday or Friday, depending on their postal code. The online survey will close on November 21, 2021, for the first portion and on December 26, 2021, for the sixth portion.

3.2 SAMPLE

3.2.1 Sampling frame and target population

The target population consists of all employees aged 15 to 74 who work in the Netherlands. The operationalization of the target population for the NEA 2021 is as follows: for the purpose of sampling, a sampling frame will be derived from the most recent Polis administration, i.e. the V2 version of March 2021, on July 16, 2021. All persons with the following characteristics will be included in the sampling frame:

1. is an employee according to the CBS derivation based on data from the Polis administration on March 26, 2021 (last Friday of March)
2. was born after September 30, 1946, and before October 1, 2006.
3. is registered as a resident in the Personal Records Database (BRP) at the time of sampling
4. belongs to a private household on July 16, 2021.

The sampling frame for the NEA 2021 contains 7.7 million individuals.

The sample frame for the sample is based on the Personal Records Database (BRP). By linking the BRP to the Policy Administration, a selection can be made of persons in the BRP who are in paid employment. The Polis Administration, managed by the UWV, contains data on all jobs of employees who are insured for employee insurance and for whom income tax is payable.

A disadvantage of using a file with a reference date that precedes the actual fieldwork is that people who started working after this reference date are missing from this file. On the other hand, the Policy Administration contains persons who left their jobs after the reference date. As a result, some of them are no longer employees at the time of the survey and will be incorrectly included in the sample. Despite these limitations, the Policy Administration provides a good approximation of the employee population in 2021.

Employees who also work as self-employed persons are also included in the sample frame. Persons who work exclusively as self-employed persons are not included in the sample frame.

3.2.2 Relocation strategy

Although samples are drawn as close as possible to the start of the observation period, it is impossible to prevent the address in the BRP from proving to be incorrect because the sample person has moved. The name and address details are checked shortly before the survey. During the observation period, people who have moved are not contacted again.

3.2.3 Sample design

For the NEA 2021, a sample of individuals is drawn from this sampling frame according to a stratified sampling design. This involves stratification by business class at SBI40 level. An overview of the business classes is given in Table B.1 of Appendix B. Business class 34 is further subdivided into strata due to an expansion of the sample in this business class. An overview of the substrata is also given in Table B.1 of Appendix B. In the sampling design, young people and persons with a non-Western migration background are overrepresented in the sample. The probability of selection for persons belonging to at least one of these groups is one and a half times greater than that for other persons. A person is considered young if he or she is 24 years of age or younger on September 30, 2021. The probability of selection for this population group is higher in order to compensate for the expected lower response rate for this group and persons with a non-Western migration background. Persons from certain business classes are also overrepresented in the sample in order to achieve at least 200 usable responses in each business class (except for SBI 40). This is the publication threshold for StatLine.

The sample size for NEA has been determined on the basis of usable responses and occupational accidents from NEA 2020. The most important principle is that the accuracy of the results at the national level must be as high as possible. A usable response is a response that is used for publication in NEA. The number of sample persons to be approached is 158,500, excluding the expansion required in strata 41 and 42. In those strata, 4,000 usable responses must be obtained. Table B.1 in Appendix B shows the samples to be drawn per stratum.

3.2.4 Sample processing

After the sample was drawn, individuals were removed from the sample whose address details were incomplete or unusable, or who lived at an address that had either already been included in a CBS sample in the previous twelve months or belonged to the institutional population. Finally, persons who were included in the NEA 2020 sample were removed from the sample. The screening dropout rate for the total population was 10.7%. The persons remaining after screening were systematically thinned out per stratum to the numbers to be selected per business class.

The total sample to be approached is divided into six equal portions by assigning the portions cyclically to the sample elements. The persons from the sample who are eligible for the conditional reward are selected at random.

For the purpose of controlling the questionnaire, additional sample data is used in the auxiliary variable SE_Cluster. Within each portion, the value of the auxiliary variable SE_Cluster is set to 1 for one

random half of the sample elements is set to 1 and for the other half to 2. A weighting factor is created for both portions (see section 3.6.4).

3.3 FIELDWORK AND RESPONSE

3.3.1 Adjustments to the research design

No adjustments were made to the research design during the fieldwork.

3.3.2 Actual response and response development

A total of 53,593 responses were achieved, representing a response rate of 31.0%. The actual response rate is therefore 0.5% higher than the response target of 30.5%. Table 3.1 shows the actual response results per sample portion.

TABLE 3.1 Response results by sample portion

SAMPLE SIZE	RESPONSE PERCENTAGE
1	30.8
2	31.2
3	31.0
4	31.0
5	32.0
6	30.3
Total response	31.0

Figure 3.1 shows the response development (%) over time (days) for 2021. Both at the start and during the fieldwork, all portions for the NEA 2021 were lower than the portions in the NEA 2020 around the same period. The response progress for all portions of NEA 2021 followed the same pattern, leading to the conclusion that the response for NEA 2021 was less successful than for NEA 2020. The fact that the response rate was lower in 2021 than in 2020 was not unique to the NEA, but was also seen in other CBS surveys conducted online.

The proportion of sample persons who logged in as a percentage of the sample sent out is 34.6%, which is 6 percentage points lower than in 2020 (40.6%). The proportion of dropouts as a percentage of logins is 10.3%, which is 0.8 percentage points higher than in 2020 (9.5%). The proportion of dropouts on a smartphone is 15.5%, which is 0.4% higher than in 2020 (15.1%).

FIGURE 3.1 Response development (%) over time (days), 2021

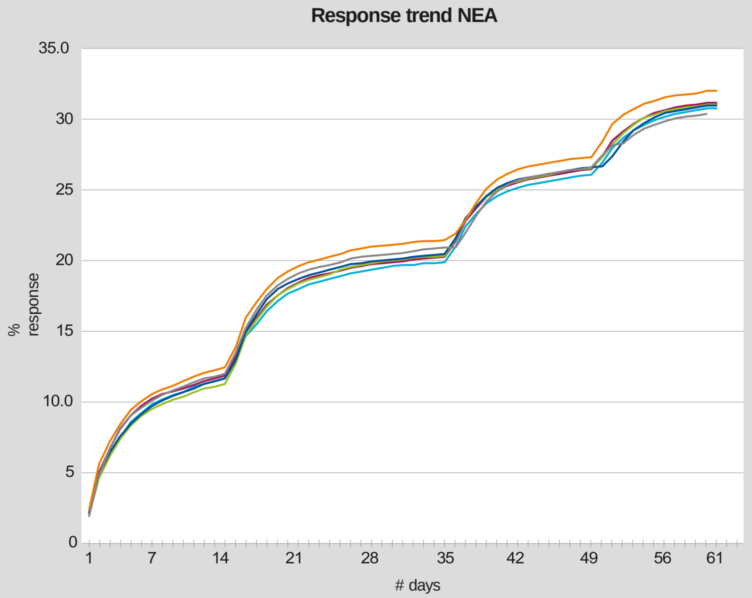

RESPONSE DEVELOPMENT	DAY 7	DAY 14	DAY 21	DAY 28	DAY 35	DAY 42	DAY 49	DAY 56	DAY 61
NEA 2021JP01	9.8	11.6	18	19.4	19.9	25.2	26.1	30.2	30.8
NEA 2021JP02	10.2	11.9	18.4	19.7	20.3	25.6	26.5	30.6	31.2
NEA 2021JP03	9.5%	11.3	18.4	19.8	20.3	25.6	26.5	30.5	31
NEA 2021JP04	9.7	11.7	18.7	20	20.5	25.7	26.6	30.4	31
NEA 2021JP05	10.6	12.5	19.6	21	21.5	26.5	27.3	31.5	32
NEA 2021JP06	10.1	12	19.1	20.3	20.9	25.7	26.6	29.8	30.3

3.3.3 Weighted response

After the survey was completed, a weighted response rate was calculated. This involves adjusting the response rate for over- and under-representation of certain groups in the sample compared to a proportionally drawn sample. This means that the response rate may be weighted according to inclusion probability, because the sample was not drawn proportionally. The weight of a sample person is equal to 1 divided by the probability with which that person was selected in the sample. In this way, persons with a higher inclusion probability count less heavily than persons with a lower inclusion probability. The inclusion weights of all individuals in the sample add up to the population total. The weighted response rate is 31.3%.

3.3.4 Usable response

Some of the responses ultimately prove to be unusable. There may be dropouts due to framework errors or insufficient completion of a completed questionnaire. Framework errors concern responses for which the date of birth and gender reported by the respondent differ from the relevant characteristics in the registration, and responses for which the respondent states that they are not an employee at the time of completion. Insufficient completion means that less than 75% of the questions that are always included in the routing have been completed with a valid answer. "Don't know" is not a valid answer, with the exception of a few questions.

Of the 172,615 people who received an invitation to participate in the survey, 53,593 responded. Of these
53,593 responses, 49,659 were usable (see Figure 3.2). The difference

is mainly among respondents who were not employed at the time of completing the survey: 3,521 responses were excluded for this reason. This corresponds to 6.6% of the responses and is lower than in the NEA 2020 (7.8%). In 2020, the percentage is higher than in previous years, and it seems likely that this is related to the coronavirus pandemic.

FIGURE 3.2 Usable response NEA 2021

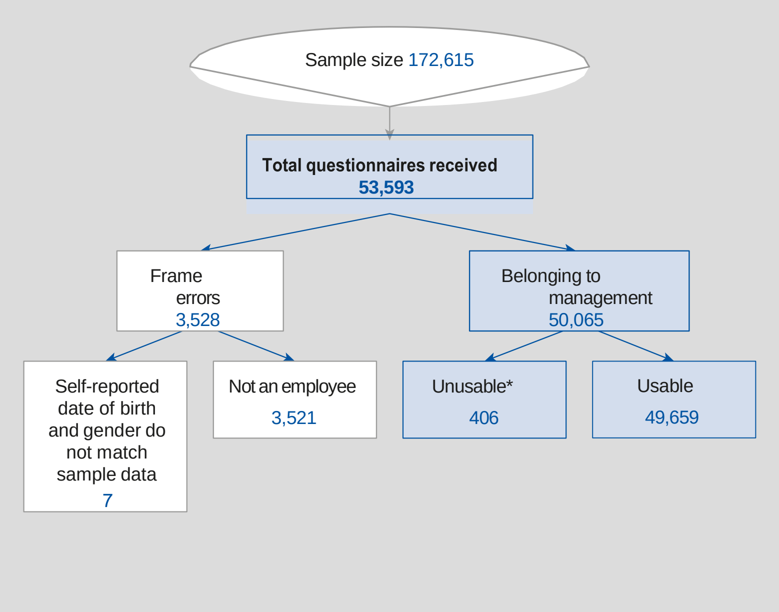

* A response is unusable if less than 75 percent of the questions that apply to all respondents on the route have been answered with a valid answer.

3.3.5 Usable responses per industry

The sample design provides for individuals from certain business classes to be overrepresented in the sample in order to achieve a minimum of 200 usable responses in each business class (with the exception of SBI 40). This is the publication threshold for StatLine. For publication purposes, a respondent does not always belong to the same industry as in the sample selection. The industry for publication purposes is characterized in the processing process on the basis of information from the questionnaire and register data with a more recent reference date than the sample data (see sections 3 .4.2 and 3 .4.3). Table B .1 in Appendix B shows the usable response per industry as known at the time of sampling and per industry for publication. In industries 2 , 6 , 11, 17, 25, and 40, there were insufficient observations for publication on StatLine.

The target of 4,000 usable responses in primary and secondary education was also not achieved.

3.3.6 Actual questionnaire duration

On average, respondents took 29.9 minutes to complete the questionnaires. This means that the average questionnaire duration decreased by 2.3 minutes compared to 2020. The starting point for questionnaire development is that the average questionnaire duration should not exceed 30.0 minutes. When determining the average questionnaire duration, all responses for which the questionnaire was started on a different date than it was completed are excluded. In addition, questionnaires are excluded from persons who have indicated that they are not employees and from persons for whom the reported date of birth and gender both deviate from the relevant characteristics in the registration. Finally, the remaining questionnaires are sorted in ascending order based on

questionnaire duration. From this sorted list, the bottom 2.5% (with the shortest questionnaire durations) and the top 2.5% (with the longest questionnaire durations) are disregarded.

3.4 DATA PROCESSING

3.4.1 Introduction

The term data processing refers to the work required to derive plausible statistical information from the answers given by respondents in the questionnaire. Data processing is automated as much as possible. A number of checks on ranges, routing, and inconsistencies were performed in accordance with the rules established by TNO and CBS. Several derived variables were then added. For variables originating from previous NEAs, the derivations from previous years from the TNO software were followed. For variables originating from CBS surveys, the CBS derivations were followed. New derivation specifications were drawn up for new variables. Furthermore, descriptions of company, occupation, and level of education attained were added to the file (see section 3.4.2), and the database was enriched with some data from the Polis Administration and the Personal Records Database (see section 3 .4.3). TNO and CBS independently performed various plausibility checks on the anonymized file containing the usable responses and then discussed their findings. In addition, they consulted with each other to draw up processing rules and derivation specifications for new variables. CBS incorporated these into the processing process.

3.4.2 Characterizing business, profession, and education

In order to determine the level of education attained by respondents, the sector in which they work, and their occupation, Statistics Netherlands (CBS) has developed a standard processing procedure for classifying companies, occupations, and education. This is in line with the CBS's standard questions for company, occupation, and education (see sections 2.2.3, 2.2.6, and 2.2.7). The level of education attained is classified entirely automatically. In most cases, the industry in which respondents work is determined on the basis of register information (Polis Administration). Under certain conditions, the industry is classified (partly automatically and partly manually), namely if the respondent is a temporary worker or seconded employee or has recently started working for their current employer. If the respondent started working for their current employer less than four months ago, the register information may still relate to the industry of their previous employer. According to the policy administration, temporary workers and seconded workers belong to the business services sector. In practice, the company to which they are sent will often be active in a different industry. The standard question for company explicitly states that it concerns the company to which the respondent has been sent. The classification process produces an SBI 2008 code. In cases where no usable code was produced, the SBI from the sample data was used. Furthermore, all respondents who answer in the Employment block that they are employed under the Social Employment Act are assigned the corresponding SBI code. A valid SBI is ultimately known for all respondents. This is used for publication. After the industry has been classified, the occupation is classified. This is done partly automatically and partly manually. The classification process uses the information collected in the Occupation block, SBI, and education level. The classification process results in an ISCO 2008 code, from which a BRC 2014 code is also derived.

3.4.3 Enrichment with register data

Because Statistics Netherlands assigns a unique serial number to each respondent in the sample, it is possible to link information from other sources (registrations) to the data from the survey.

The NEA database is enriched with the following characteristics:
1) gender, 2) age, 3) industry, 4) migration background, 5) age of youngest child, 6) household composition, 7) position in the household, 8) municipality code, 9) region, 10) urbanity, 11) collective labor agreement sector, 12) whether or not a regular collective labor agreement applies, 13) incapacity for work, and 14) income and prosperity.

The final values for gender, age, and industry in the file are not based exclusively on register data. At the beginning of the questionnaire, the gender and date of birth from the register are verified. The respondent can change one of these two pieces of information, but not both. If the respondent indicates that both pieces of information are incorrect, the questionnaire ends and the response is not considered usable. If the respondent changes one of the two pieces of information, the data from the questionnaire are used as the final data for weighting and publication.

Two values per respondent are also available for industry in the processing process. The industry according to classification (see section 3.4.2) is used for publication. The industry according to the sample data, derived from the Policy Administration, is used for weighting. Since the classification process uses data from the Policy Administration, the two values generally correspond. The difference between the two variables is particularly relevant for business services and temporary agency workers. In the Policy Administration, temporary agency workers belong to business services. In the classified varia-

business services and temporary workers. In the Policy Administration, temporary workers belong to business services. In the classified variable, temporary workers have the SBI of the industry to which they are assigned. In practice, this will often be an industry other than business services.

3.5 COMPARISON OF RESPONSE WITH EXPANDED SAMPLE

Because different groups of people in the sample respond more or less frequently, the response is less representative than the sample. If we compare the distribution of a number of background characteristics (gender, age, migration background, educational level, industry, urbanity, and region) between the respondents of the NEA and the sample, it becomes clear in which direction and to what extent the representativeness is distorted for these characteristics. The conclusions of these comparisons are described below. The corresponding tables
B.2 to B.8 are shown in Appendix B.

The distribution of men and women in the NEA response and in the sample shows that proportionally more women participated in the NEA (51% in the response) compared to the sample (49% in the sample) and fewer men (49% in the response compared to 51% in the sample). A comparison of the percentage size of age categories between the response and the sample shows that men aged 55 to 65 respond more often; the difference is 9 percentage points. Men in the 45 to 55 age category are overrepresented in the response by 3 percentage points due to relatively low non-response. Men in the 15 to 45 age group respond less well in the NEA

. As with men, women in the 45 to 65 age group also respond better. Women aged 15 to 45 are underrepresented in the response.

The composition of the NEA response and the sample in terms of gender and migration background show that people with a Dutch background respond relatively often, both men and women. People with a non-Western migration background respond relatively less often than people with a Western or Dutch background.

A slightly different procedure was used to compare the distribution of responses by educational level. In this specific case, the distribution of NEA responses by educational level attained – adjusted for the different chances of respondents being selected – is compared with the Polis Administration. The distribution by educational level from the 2020 Labor Force Survey (EBB) has been applied to the latter source. This means that for each subpopulation in the Polis Administration, the composition by educational level from the EBB is taken over according to gender and age (six 10-year age groups). The reason for this different procedure for educational level is that the Polis Administration does not contain any information about educational level. The comparison shows that, in proportion, more people with higher vocational education respond in the NEA compared to the composition in the EBB. Employees with a university education also respond relatively more often in the NEA. Among employees with a lower level of education than higher vocational education, the willingness to respond in the weighted NEA response is actually lower.

Finally, the composition of the NEA response and the sample were examined in terms of industry, urbanization, and region. The composition of the NEA response by industry shows that virtually all 40 industries are well represented. Employees in 1) rental of movable property and other business services and 2) accommodation, food and beverage services respond less often in the NEA; compared to the sample, the share in these industries is more than one percentage point lower. Respondents working in 1) general secondary education and 2) primary and special education respond above average in the NEA.

In terms of urbanity and provinciality, the NEA response accurately reflects the sample. The exception to this is highly urban areas, which account for 3 percentage points less in the response than in the sample. The shares of Amsterdam, Rotterdam, and The Hague in the regional distribution also lag slightly behind in the response, all three by 1 percentage point.

3.6 WEIGHTING

Differences between the target population and the NEA response—for example, in the distribution of men and women and of young and older employees—can reduce the representativeness of NEA findings. To minimize bias due to selectivity in the response, the response is weighted. The sampling already took into account a lower response rate among young people and people with a non-Western migration background by increasing the sample fraction for these population groups (oversampling). Furthermore, the sample is distributed in such a way that the expected response per industry is proportional to the size of the industry, and some industries are oversampled

in order to make more accurate estimates. If, despite or precisely because of this oversampling, there is an uneven distribution between the response and the target population, this is corrected for by weighting.

The application of weights is referred to as weighting if the weights add up to the population size (according to the sampling frame). These weights are called weighting weights. Analyses often also use weights that are standardized in such a way that the average of the weights is 1. In that case, the sum of the weights corresponds to the number of respondents.

The starting point for the weighting model of the NEA 2021 is the weighting model of the NEA 2020 (Hooftman et al., 2021). The following section briefly describes the method used to determine weights.

3.6.1 Determining the weights

The NEA uses unequal sampling and there are differences in response rates. As a result, the composition of the response will differ from the composition of the population. The basic technique that can remedy this is post-stratification. In post-stratification, the population is divided into strata, for example age categories. Each person within a stratum is assigned a weight such that the sum of the weights within the stratum is equal to the population total within the same stratum.

At the NEA, a number of stratifications are applied consecutively. First, the weight for the first stratum is determined. This weight serves as the starting point for the next stratum. The weights are adjusted if necessary necessary to arrive at the population total of the next stratum. This procedure is repeated until all stratifications have been completed. This is the end of the first iteration. Then a second iteration begins, in which all stratifications are repeated.

In general, the weights converge to final weights where the estimated stratum totals for all stratifications correspond to the predetermined population total. This method is known as IPF (Iterative Proportional Fitting) and RR estimation (Raking Ratio estimation). This method can be applied using the BASCULA weighting program, part of BLAISE, which was developed by Statistics Netherlands (CBS).

3.6.2 The stratifications and background data used

The NEA response is weighted according to the following variables:

- gender
- age in six 10-year age groups
- industry in 44 sections and subsections of the standard industrial classification (SBI2008)
- migration background in five categories: persons with a Dutch background, and persons with a Western and non-Western background, with the latter two groups divided into first and second generation
- region: the provinces and the four largest municipalities separately
- urbanity in five classes
- level of education attained in five classes.

The first six variables are taken from the sampling frame (the Polis administration). The stratum classification therefore comes directly from the sampling frame.

. As in previous years, the variables gender and age have been corrected for self-reporting. The variable industry has not been corrected for self-reporting (see section 3.4.3).

The variable educational attainment does not originate from the sample frame. The Policy Administration does not contain any information about educational attainment. The stratum distribution of the variable educational attainment is based on the results of the 2020 Labor Force Survey (EBB). A limitation of this is that neither the NEA nor the EBB has information about the educational attainment of all respondents. For the purposes of weighting, these persons are added to the Primary Education category. In addition, the EBB is based on a sample from the entire population and an estimate is made of the distribution of employees aged 15 to 75 across categories of educational attainment. The NEA, on the other hand, is based on a sample of employees registered in the Polis administration. These differences mean that the weighting to the distribution of educational attainment levels from the EBB may introduce a slight deviation.

The following stratifications were applied to determine the weighting coefficients:

- gender x age group x migration background
- industry
- region x urbanity
- gender x age group x level of education attained

The x sign indicates that the strata are formed by combinations of the variables shown.

3.6.3 The weights

Table B.9 shows the final weights of the six age groups for men and women. For all men combined, they are neither over- nor under-represented in the NEA compared to the sampling frame[1]. They therefore have a weighting coefficient of approximately 1 . Men aged 55 to 75 and women aged 55 to 65 are particularly over-represented in the response and therefore have a relatively low weighting. Men aged 25 to 35 are the most underrepresented in the response of all age groups and therefore have the highest weighting coefficient on average.

The final weights for the classification by migration background and gender are shown in Table B.10. For both men and women, individuals with a first-generation Western migration background and individuals with a second-generation non-Western background have the highest average weighting coefficients. They are the most underrepresented. Women with a Dutch background have the lowest weighting coefficient. Persons with an unknown migration background are classified for weighting purposes as persons with a Dutch background.

Compared to the EBB, employees with primary education or a mavo/mbo diploma as their highest level of education are underrepresented in the NEA (Table B.11). They have a weighting coefficient that is significantly higher

1 This seems to contradict what is stated in section 3.5, namely that men are underrepresented in the response compared to the sample drawn. The fact that men are ultimately not underrepresented compared to the sample frame is because a slight overrepresentation of men arose when the sample was drawn. This overrepresentation is offset by the fact that men respond slightly less than women.

than 1. Among the NEA respondents, employees with a higher professional education diploma and academics are overrepresented. Most industries are underrepresented in the response (Table B.12). In contrast, in ten industries the weighting coefficient is less than 1 . These are all industries that are overrepresented in the sample design in order to obtain a sufficient response for publication.

The national coverage of the NEA appears to be good. The weighting coefficients for the classification by urbanity (Table B.13) and region (Table B.14) are close to 1. The fact that three of the four largest municipalities have weights well above 1 is related to the fact that young people and people with a non-Western background are underrepresented in the response. These two groups are more likely than others to live in large cities.

It is concluded that the NEA response reasonably well reflects the sampling frame in terms of gender, age, migration background, and industry. This is partly due to the oversampling of groups of people who are relatively unresponsive, such as young people and people with a non-Western migration background. In terms of urbanization and region, the NEA response closely matches the sampling frame. The NEA weighting model includes demographic, socioeconomic, and regional characteristics. These characteristics are related to both the response and working conditions in a broad sense (the main topic of the NEA). The weighting ensures that the distribution of the weighting variables in the response corresponds to the distribution of those same variables in the sampling frame.

3.6.4 Weighting split-half design

In the NEA 2021, the weighting model was not only applied to all usable responses, but also separately to the responses in the two random portions (see section 3.2.4). There are therefore three weighting factors in total: one for the entire sample, one for portion 1 , which was asked questions about the climate in the workplace, and one for portion 2, which was asked questions about the value of work. This means that, based on observations in the half sample, it is still possible to publish data on all employees.

The procedure, stratifications used, and background data are the same as described in sections 3.6.1 and 3.6.2. The NEA weighting model was developed in the past for a smaller sample size than that planned for 2021 and can therefore be applied to two clusters in 2021 without modification.

The final weights for the two portions are also comparable to the final weights described in section 3.6.3 for the entire sample. Table B.15 shows the final weights of the six age groups of men and women for both portions. As in both portions combined (see Table B.9), men are not over- or underrepresented in relation to the sample frame in either portion. Their weighting coefficient is therefore approximately equal to 1 . Men aged 55 to 75 and women aged 55 to 65 are particularly overrepresented in the response of both portions and therefore have a weight well below 1. Men aged 25 to 35 are the most underrepresented of all age groups in the response of both portions and therefore have the highest weighting coefficient on average.

3.7 PROTECTION OF PERSONAL DATA

Statistics Netherlands is responsible for collecting data for the NEA and also processes personal data in this process. Personal data is information that relates to a person or can be traced back to that person. Examples include a name, home address, or income. An email address is also personal data. Statistics Netherlands handles respondents' data with care and secures it using technical and organizational measures. The most important measures are:

- The information that respondents enter via the internet is sent to CBS in encrypted form. At CBS, the data is stored in a secure environment. Only authorized employees have access to this data.
- As early as possible in the process, CBS removes directly identifiable personal data from the files. This means that research files never contain data such as names, addresses, or citizen service numbers.
- CBS employees must comply with the confidentiality obligation laid down in the CBS Act. All employees have signed a confidentiality agreement.
- CBS uses the data solely for statistical and scientific purposes. Use for fiscal, administrative, control, and judicial purposes is prohibited by law. CBS also does not use the data for marketing purposes.
- CBS has its own Data Protection Officer. This officer checks whether CBS handles personal data with care. He keeps a register of all processing of personal data. The NEA is included in this register.
- CBS complies with the General Data Protection Regulation (GDPR). This regulation serves to protect the privacy of citizens. CBS also complies with the privacy provisions in the CBS Act, the European Statistical Regulation (Statistical Law), the Code of Practice for European Statistics, and its own code of conduct.
- The Dutch Data Protection Authority supervises compliance with the legal rules for the protection of personal data.

Potential respondents have been informed about the safeguarding of their privacy in the letters and reminders and on the CBS website.

Apart from CBS, only TNO and SZW have access to the NEA microdata file. TNO and SZW also comply with the privacy provisions in the CBS Act and the GDPR. CBS makes microdata available to research institutions under strict conditions via the Remote Access environment at CBS. CBS checks the output for traceability. In publications based on the NEA, individuals are not recognizable or traceable.
