# Supplementary material for: Testing psychosocial work adversities as a necessary condition for work-related emotional exhaustion in young workers: a cross-sectional necessary condition analysis on a national general working population-based survey
Source: BMJ Open. 2025 Nov 12;15(11):e094485. doi: 10.1136/bmjopen-2024-094485 (PMC12612735; doi:10.1136/bmjopen-2024-094485)
Supplement: online supplemental file 2 [file bmjopen-15-11-s002.pdf]

Supplementary Information.

Table 1. Overview of all variables, origin, sample items, response categories and internal reliability

**Outcome variable**				
**Adverse mental health**	**Origin (see note)**	**Items**	**Response category**	**Internal reliability (Cronbach's alpha)***
Emotional Exhaustion<br>(5 items)	UBOS (1)	1. I feel emotionally drained by my work.<br>2. At the end of a workday, I feel empty.<br>3. I feel tired when I get up in the morning and face my work.<br>4. It takes a lot out of me to work with people all day.<br>5. I feel completely exhausted by my work.	(1) Never<br>(2) Several times a year<br>(3) Monthly<br>(4) Several times a month<br>(5) Every week<br>(6) Several times a week<br>(7) Every day	0.89
**Exposure variables**				
**Composite Psychosocial work adversities (28 items)**				
The combination of high job demands and low job resources				
**Composite Job demands (17 items)**	**Origin (see note)**	**Item**	**Response category**	
Quantitative demands<br>(3 items)	JCQ (2)	1. Do you have to work very quickly?<br>2. Do you have a lot of work to do?<br>3. Do you have to work extra hard?	(1) Never<br>(2) Sometimes<br>(3) Often<br>(4) Always	0.84
Emotional demands<br>(3 items)	COPSOQ (3)	1. Does your work put you in emotionally difficult situations?<br>2. Is your work emotionally demanding?<br>3. Does your work affect you emotionally?	(1) Never<br>(2) Sometimes<br>(3) Often<br>(4) Always	0.84
Cognitive demands<br>(3 items)	JCQ (2)	1. Does your work require intensive thinking?<br>2. Does your work require you to concentrate?<br>3. Does your work demand a lot of your attention?	(1) Never<br>(2) Sometimes<br>(3) Often<br>(4) Always	0.77
Internal inappropriate conduct<br>(4 items)	European Survey (4)	Can you indicate the extent to which you have personally experienced in the past 12 months:<br>1. unwanted sexual attention by managers or colleagues?<br>2. intimidation by managers or colleagues?<br>3. harassment by managers or colleagues?<br>4. bullying by managers or colleagues?	(1) No, never<br>(2) Yes, sometimes<br>(3) Yes, often<br>(4) Yes, very often	Not applicable (see methods)

External inappropriate conduct (4 items)	European Survey (4)	Can you indicate the extent to which you have personally experienced in the past 12 months:<br>1. unwanted sexual attention by clients (patients, students, or passengers, etc.)?<br>2. intimidation by clients (patients, students, or passengers, etc.)?<br>3. harassment by clients (patients, students, or passengers, etc.)?<br>4. bullying by clients (patients, students, or passengers, etc.)?	(1) No, never<br>(2) Yes, sometimes<br>(3) Yes, often<br>(4) Yes, very often	Not applicable (see methods)
**Composite Job resources (11 items)**	**Origin (see note)**	**Item**	**Response category**	
Autonomy/<br>decision latitude<br>(6 items)	JCQ (2),<br>POLS (5),<br>NWCS (6)	1. Can you make your own decisions on how to execute your work?<br>2. Do you determine the order of your tasks yourself?<br>3. Can you set your own pace of work?<br>4. Do you have to come up with solutions yourself to do certain things in your work?<br>5. Can you take leave whenever you want?<br>6. Can you decide for yourself what hours you work?	(1) Yes, regularly<br>(2) Yes, sometimes<br>(3) No	0.75
Colleague support (2 items)	JCQ (2)	1. My colleagues take a personal interest in me.<br>2. My colleagues are friendly.	(1) Completely disagree<br>(2) Disagree<br>(3) Agree<br>(4) Completely agree	0.80
Manager support (2 items)	JCQ (2)	1. My manager cares about the well-being of employees.<br>2. My manager pays attention to what I say	(1) Completely disagree<br>(2) Disagree<br>(3) Agree<br>(4) Completely agree	0.85
Development opportunities (1 item)	NWCS (6)	1. Does your supervisor encourage the development of your knowledge and skills?	(1) No<br>(2) Yes, to a limited extent<br>(3) Yes, to a large extent	Not applicable (see methods)

Note. (1) Subscale of Utrecht Burnout Scale (UBOS [13]); which is an adjusted Dutch version of the Maslach Burnout Inventory-General Survey (MBI-GS [14]); (2) Job Content Questionnaire (JCQ [15]); (3) Copenhagen Psychosocial Questionnaire (COPSOQ [16]); (4) Based on European Survey of the European Foundation for the Improvement of Working and Living conditions [17]; (5) Permanent Onderzoek Leef Situatie (POLS, Statistics Netherlands (CBS)) as described in NWCS [12]; (6) One item on 'time autonomy' within the autonomy scale was constructed specifically for the NWCS [12]; one item on development opportunities was constructed specifically for the NWCS[12].
* Cronbach's alphas are calculated for the subsample used in this study.

Table 2. Bottleneck tables, based on CE-FDH line, after outlier exclusion.

Work-related Emotional Exhaustion*	H1: Composite **job demands and job resources**	H2: Job demands in **low job resource** group	H2: Job demands in **high job resource** group
1	NN**	NN**	NN**
1,2	NN**	0.022	0.022
1,4	0.049	0.022	0.044
1,6	0.049	0.022	0.044
1,8	0.049	0.022	0.044
2	0.071	0.022	0.044
2,2	0.083	0.022	0.067
2,4	0.083	0.022	0.067
2,6	0.083	0.022	0.067
2,8	0.083	0.022	0.067
3	0.083	0.022	0.067
3,2	0.083	0.022	0.067
3,4	0.083	0.044	0.067
3,6	0.083	0.044	0.067
3,8	0.083	0.044	0.067
4	0.117	0.089	0.111
4,2	0.148	0.089	0.111
4,4	0.148	0.089	0.111
4,6	0.148	0.089	0.111
4,8	0.148	0.089	0.156
5	0.148	0.089	0.156
5,2	0.148	0.111	0.200
5,4	0.148	0.111	0.200
5,6	0.148	0.111	0.200
5,8	0.148	0.111	0.200
6	0.148	0.111	0.200
6,2	0.148	0.200	0.267
6,4	0.262	0.200	0.356

6,6	0.318	0.222	0.356
6,8	0.318	0.378	0.356
7	0.343	0.378	0.400

* 1=never; 2= several times a year; 3=monthly; 4=several times a month; 5=every week; 6=several times a week; 7=every day.

** NN: not necessary, meaning that for a level of 1 (never) on work-related emotional exhaustion (Y) no minimum level of psychosocial work adversity (X) is required.

Table 3. Robustness checks for composite high job demands and low job resources as necessary condition.

Year	N	Outliers	Effect size	p-value	Outlier Count
2014	4,071	included	0.15	<.001	NA
2014	4,070	excluded	0.10	<.001	1
2015	5,390	included	0.12	<.001	NA
2015	5,390	excluded	0.12	<.001	0
2016	5,281	included	0.09	<.001	NA
2016	5,281	excluded	0.09	<.001	0
2017	5,199	included	0.15	<.001	NA
2017	5,199	excluded	0.15	<.001	0
2018	7,354	included	0.14	<.001	NA
2018	7,354	excluded	0.14	<.001	0
2019	6,944	included	0.08	<.001	NA
2019	6,944	excluded	0.08	<.001	0
2020	6,782	included	0.10	<.001	NA
2020	6,782	excluded	0.10	<.001	0
2021	5,791	included	0.11	<.001	NA
2021	5,791	excluded	0.11	<.001	0

Table 4. Robustness checks for composite job demands as necessary condition contrasted for high versus low job resources.

Year	N[1]	Outliers	High or low job resource	Effect size	p-value	Outlier count
2014	4,264	Included	All	0.05	.216	NA
	1,939		Low job resource	0.06	.039	NA
	2,132		High job resource	0.12	.089	NA
	4,263	Excluded	All	0.06	.029	1
	1,938		Low job resource	0.08	.002	1
	2,132		High job resource	0.12	.089	0
2015	5,627	Included	All	0.07	<.001	NA
	2,612		Low job resource	0.08	.001	NA
	2,778		High job resource	0.19	<.001	NA
	5,626	Excluded	All	0.10	<.001	1
	2,611		Low job resource	0.11	<.001	1
	2,778		High job resource	0.19	<.001	0
2016	5,536	Included	All	0.05	0.018	NA
	2,632		Low job resource	0.07	.001	NA
	2,649		High job resource	0.12	.005	NA
	5,535	Excluded	All	0.07	<.001	1
	2,631		Low job resource	0.08	<.001	1
	2,649		High job resource	0.12	.005	0
2017	5,465	Included	All	0.10	<.001	NA
	2,495		Low job resource	0.10	<.001	NA
	2,704		High job resource	0.17	<.001	NA
	5,465	Excluded	All	0.10	<.001	0
	2,495		Low job resource	0.10	<.001	0
	2,704		High job resource	0.17	<.001	0
2018	7,744	Included	All	0.01	.658	NA
	3,675		Low job resource	0.01	.749	NA
	3,679		High job resource	0.12	<.001	NA
	7,742	Excluded	All	0.06	<.001	2
	3,673		Low job resource	0.06	<.001	2
	3,679		High job resource	0.12	<.001	0
2019	7,281	Included	All	0.06	<.001	NA
	3,394		Low job resource	0.06	<.001	NA
	3,550		High job resource	0.11	<.001	NA
	7,281	Excluded	All	0.06	<.001	0
	3,394		Low job resource	0.06	<.001	0
	3,550		High job resource	0.11	<.001	0
2020	7,050	Included	All	0.10	<.001	NA
	3,349		Low job resource	0.11	<.001	NA
	3,433		High job resource	0.15	<.001	NA
	7,050	Excluded	All	0.10	<.001	0
	3,349		Low job resource	0.11	<.001	0

	3,433		High job resource	0.15	<.001	0
2021	6,074	Included	All	0.07	<.001	NA
	2,885		Low job resource	0.07	<.001	NA
	2,906		High job resource	0.15	<.001	NA
	6,073	Excluded	All	0.10	<.001	1
	2,884		Low job resource	0.10	<.001	1
	2,906		High job resource	0.15	<.001	0

Note[1]: N's from "low" and "high" job resources rows do not add up to N from "all" row due to missing data on job resources.

Figure 1. Scatterplot for composite high job demands given quartiles of job resources (x) and work-related emotional exhaustion (y) without outliers

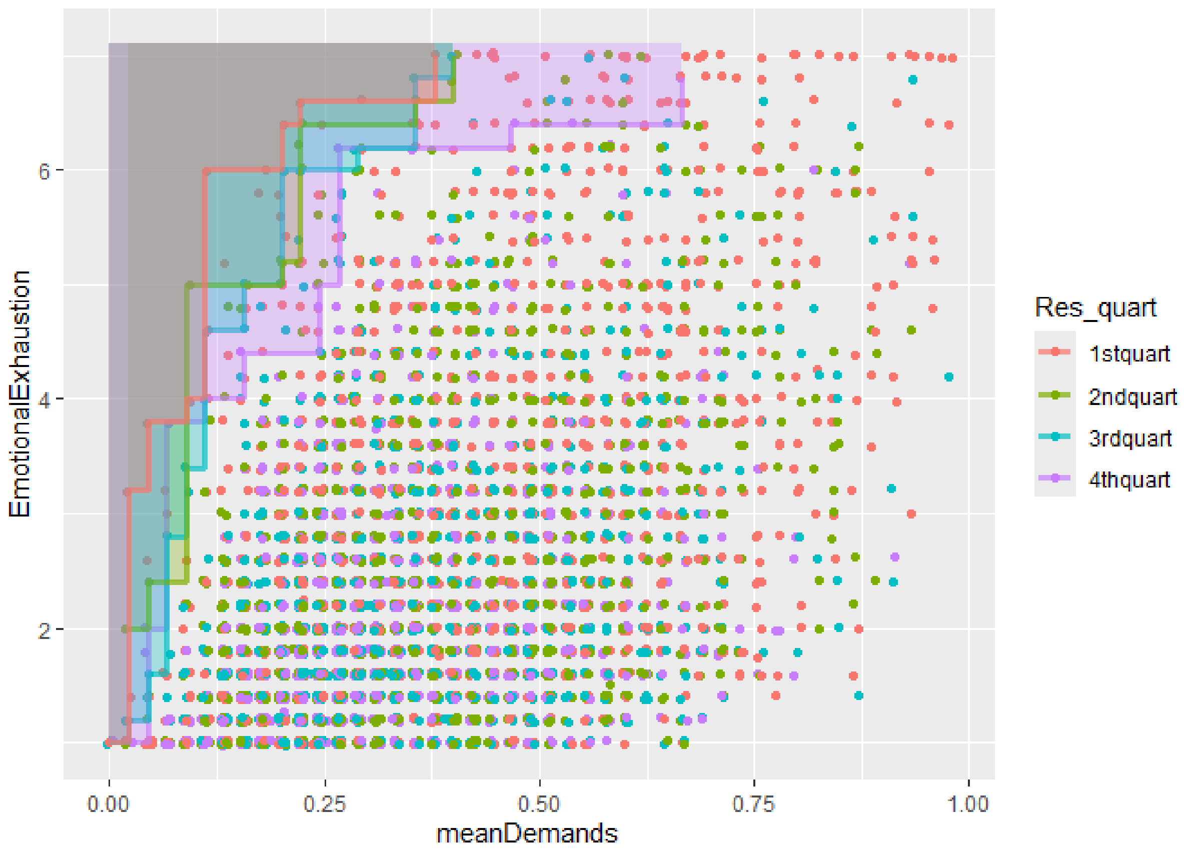

Note. The stepwise ceiling envelopment lines depicted are CE-FDH lines.
