# Supplementary material for: Testing psychosocial work adversities as a necessary condition for work-related emotional exhaustion in young workers: a cross-sectional necessary condition analysis on a national general working population-based survey
Source: BMJ Open. 2025 Nov 12;15(11):e094485. doi: 10.1136/bmjopen-2024-094485 (PMC12612735; doi:10.1136/bmjopen-2024-094485)
Supplement: Supplementary file 3 [file bmjopen-2024-094485.reviewer_comments.pdf]

PEER REVIEW HISTORY

BMJ Open publishes all reviews undertaken for accepted manuscripts. Reviewers are asked to complete a checklist review form and are provided with free text boxes to elaborate on their assessment. These free text comments are reproduced below.

ARTICLE DETAILS

Title (Provisional)

Testing psychosocial work adversities as a necessary condition for work-related emotional exhaustion in young workers: a cross-sectional necessary condition analysis on a national general working population based survey

Authors

Schelvis, Roosmarijn MC; van Veen, Malte; Tamminga, Sietske J; Oude Hengel, Karen; Nieuwenhuijsen, Karen; Boot, Cécile; Dul, Jan

---

VERSION 1 - REVIEW

Reviewer 1

Name Liu, Xiaolei

Affiliation Department of Neurology, The First Affiliated Hospital of Kunming Medical University

Date 26-Nov-2024

COI None

Abstract

Design, outcomes and methods

The authors should mention the study period, how this survey was conducted (face-to-face interview, flyer questionnaire distribution or distributed by an app through smartphones), and where the participants came from (Did randomised sampling or simple sampling select them?). As I mentioned in the method section, this method is in Dutch, detailed information in English could help readers to understand this research better.

Besides, the sex ratio of all participants should be mentioned.

Results

Key results should be mentioned together with data, not only descriptive sentences, e.g. effect size, 95% CI of ES, p-value.

Conclusion

The first sentence in the Results section and the last sentence in the Conclusion section are relevant to the conclusion.

Method

Study population & ethics approval

The following absences should be added

1. The inclusion and exclusion criteria
2. The diagram follows the participants
3. The approved number
4. Questionnaire recovery rate and valid questionnaire recovery rate

As the relevant reference (van Dam L, Mars G, Knops J, et al. Nationale Enquête Arbeidsomstandigheden 2021) of the 2021 wave of the Netherlands Working Condition Survey (NWCS) was in Dutch, an English version of the explanation is needed in the submitted manuscript.

Outcome variable: work-related emotional exhaustion

I suggest moving Table 1 to the Appendix and listing all the items this table mentioned.

In lines 144-149, the authors mentioned

Work-related emotional exhaustion was measured using the validated Utrecht Burnout Scale [13], an adjusted Dutch version of the Maslach Burnout Inventory-General Survey (MBI-GS)[14]. The subscale consisted of five items (e.g. "I feel emotionally exhausted by my work") and the seven-point response scale ranged from (1) "never" to (7) "every day" (see Table 1). The work-related emotional exhaustion score was calculated as the mean score of the items (range 1 to 7), with higher scores indicating more emotional exhaustion.

How many items need to be calculated to get the mean score? 5, 7, or 5+7?
I suggest the author submit the relevant scale (Utrecht Burnout Scale) in the Appendix to help me understand this research.

Data analysis

Sample size calculation or estimation should be added to reflect the Hypotheses.

Results

Table 2

What does the word 'range' mean? Min-Max, or Q1-Q3?

Educational level: what are the definitions of Low, Intermediate, and High?

Table 3

Does those NN in the 'Composite job demands' column refer to zero? Please provide the numbers the authors can obtain from the analysis results.

Effect size

I suggest that all the effect size (ES) reported in this section should be followed by their 95% CI of relevant ES.

Ethics approval

In lines 380-382, please add the approval number.

**Reviewer**	**2**
**Name**	**Feher, Gergely**
**Affiliation**	**University of Pécs, Centre for Occupational**
**Date**	**05-Jul-2025**
**COI**	**None**

This manuscript offers an innovative and valuable contribution to occupational health research by applying Necessary Condition Analysis (NCA) to assess the role of psychosocial work adversities in emotional exhaustion among young workers. The paper is theoretically well-grounded, methodologically rigorous, and highly relevant to both academic and policy audiences. I commend the authors for the clarity of writing, the robustness of their findings, and the thoughtful interpretation of results.

Major Strengths

Novel Methodological Approach:

The use of NCA introduces a fresh analytical lens to occupational mental health research. By focusing on necessary (rather than probabilistic) conditions, the study adds a deterministic perspective to the literature, which has largely been dominated by average-effect models such as regression or SEM. The authors also correctly emphasize the practical utility of NCA in formulating policy thresholds.

Robustness of Results:

The main findings are replicated across seven waves of nationally representative survey data (2014–2020), thereby greatly enhancing the external validity and credibility of the conclusions.

Theoretical Integration:

The manuscript is well anchored in the Job Demands–Resources (JDR) model, which is appropriately adapted to the context of necessity logic. The concept of equifinality is correctly invoked to justify composite score usage.

Clear Policy Implications:

The findings suggest that without psychosocial adversity, high levels of emotional exhaustion are virtually absent. This is a powerful message for occupational health policy and workplace design.

Major Concerns

Cross-sectional Design and Causality:

Although the authors acknowledge this limitation, it is worth reiterating that the cross-sectional nature of the study limits causal inference, even under necessity logic. It is important to explicitly state that NCA can detect statistical necessity, but not causal necessity without temporal precedence.

Operationalization of High vs. Low Resources:

The median split used to dichotomize job resources (for hypothesis 2) may reduce statistical power and introduce arbitrary classification. Sensitivity analyses using alternative cut-offs (e.g., tertiles or quartiles) could enhance the robustness of the subgroup comparisons.

Interpretability of Composite Scores:

While the composite indices are theoretically justified, the practical interpretation of what constitutes a “high” or “low” adversity score (e.g., 0.15) may be ambiguous for readers or policymakers. The discussion would benefit from more concrete examples or thresholds.

Minor Points and Recommendations

Please clarify in the Methods whether Cronbach’s alphas were calculated on the study sample or obtained from prior validation studies.

The bottleneck tables are informative, but their layout could be improved for readability (e.g., alignment and value formatting).

The rationale for using the CE-FDH ceiling line could be briefly elaborated with references or figures for readers unfamiliar with the method.

Consider including a figure summarizing the conceptual model (e.g., hypothesized necessity logic within the JDR framework).

VERSION 1 - AUTHOR RESPONSE

Reviewer: 1
Dr. Xiaolei Liu, Department of Neurology, The First Affiliated Hospital of Kunming Medical University
Comments to the Author:

R.1.1 Abstract
Design, outcomes and methods
The authors should mention the study period, how this survey was conducted (face-to-face interview, flyer questionnaire distribution or distributed by an app through smartphones), and

where the participants came from (Did randomised sampling or simple sampling select them?). As I mentioned in the method section, this method is in Dutch, detailed information in English could help readers to understand this research better.
Besides, the sex ratio of all participants should be mentioned.

We would like to thank the reviewer for the time, attention and effort devoted to reviewing our work.

We have clarified in the abstract that we performed a secondary analysis on an existing national working population based dataset. In the abstract, with its limited word count, we've focused on the description of the subsample of young workers we've used in the current study. We've added some more information about the subsample, i.e. the sex ratio of participants.

For brevity in the paper, we've added the remaining requested information regarding the dataset to a separate supplementary file. It contains an AI-generated translation in English of chapter 3 (Data collection and processing) of the report on the Netherlands Working Conditions Survey 2021, which was indeed only available in Dutch. In this file, the interested reader can learn a.o. that the original survey was conducted via computer assisted web interviewing (cawi) and that a stratified sampling procedure was followed.

R.1.2 Abstract - Results
Key results should be mentioned together with data, not only descriptive sentences, e.g. effect size, 95% CI of ES, p-value.

We've rewritten the results section of the manuscript to include the effect size and p-value (95% CI's are not used in NCA), it now reads:

> **A high level of the composite on job demands and job resources is necessary for a high level of work-related emotional exhaustion in young workers (effect size = 0.11, p-value < .001), and the same applies to the composite score of high job demands alone (effect size = 0.10, p < .001). The necessity threshold for job demands, which guarantees the absence of a particularly high level of work-related emotional exhaustion, was higher for the group of young workers with high job resources compared to young workers with low job resources.**

R.1.3 Abstract - Conclusion
The first sentence in the Results section and the last sentence in the Conclusion section are relevant to the conclusion.

We've rewritten the conclusion according to the suggestion and it now reads:

> **Both psychosocial work adversities were necessary conditions for high work-related emotional exhaustion in young workers. The necessity threshold for job demands was higher for young workers with high job resources, compared to the group with low resources. This indicates that removing psychosocial work adversities and ensuring the presence of job resources might contribute to the prevention of high work-related emotional exhaustion in young workers.**

R.1.4 Method
Study population & ethics approval
The following absences should be added
1. The inclusion and exclusion criteria

2. The diagram follows the participants
3. The approved number
4. Questionnaire recovery rate and valid questionnaire recovery rate >> response rate?
As the relevant reference (van Dam L, Mars G, Knops J, et al. Nationale Enquête Arbeidsomstandigheden 2021) of the 2021 wave of the Netherlands Working Condition Survey (NWCS) was in Dutch, an English version of the explanation is needed in the submitted manuscript.

All requested information relates to the source file this secondary data analysis draws from, therefore this information has been added to the supplementary file with an English translation of the Dutch methodological report on the Netherlands Working Conditions Survey 2021.

R.1.5 Outcome variable: work-related emotional exhaustion
I suggest moving Table 1 to the Appendix and listing all the items this table mentioned.

We have done so accordingly.

R.1.6
In lines 144-149, the authors mentioned

Work-related emotional exhaustion was measured using the validated Utrecht Burnout Scale 13, an adjusted Dutch version of the Maslach Burnout Inventory-General Survey (MBI-GS)14. The subscale consisted of five items (e.g. “I feel emotionally exhausted by my work”) and the seven-point response scale ranged from (1) “never” to (7) “every day” (see Table 1). The work-related emotional exhaustion score was calculated as the mean score of the items (range 1 to 7), with higher scores indicating more emotional exhaustion.
How many items need to be calculated to get the mean score? 5, 7, or 5+7?

We can imagine this formulation has led to unclarity. The scale consists of five items, the response scale ranged from 1 to 7. The mean score has been calculated by adding the scores on the separate items and dividing them by five. We have deleted ‘range 1 to 7’, the sentence now reads:

> **The subscale consisted of five items (e.g. “I feel emotionally exhausted by my work”) and the seven-point response scale ranged from (1) “never” to (7) “every day” (see Table 1). The work-related emotional exhaustion score was calculated as the mean score of the items, with higher scores indicating more emotional exhaustion.**

R.1.7.
I suggest the author submit the relevant scale (Utrecht Burnout Scale) in the Appendix to help me understand this research

In line with comment R.1.5 on the outcome variable, the version of the scale used in this study, which is based on the Utrecht Burnout Scale, can now be found in table 1 of the supplementary information.

R.1.8 Data analysis
Sample size calculation or estimation should be added to reflect the Hypotheses.

For this secondary analysis for a subsample on young workers, we did not perform a sample size calculation. For the estimate of the required sample in the source dataset (Netherlands Working Conditions Survey 2021) we kindly refer to the new supplementary file with the translation of the methodological approach.

R.1.9. Results - Table 2
What does the word 'range' mean? Min-Max, or Q1-Q3?

This has been clarified for the two variables concerned, that is: age (min 18.0; max 29.0) and working hours per week (min 17.0; max. 95.0)

R.1.10 Educational level: what are the definitions of Low, Intermediate, and High?
Educational level was defined according to the definition of the Central Bureau of Statics Netherlands, the specifics are added as a footnote to table 1. The label of the educational levels is also adjusted accordingly (low > elementary; intermediate > vocational; high > academic)

> **[b] Elementary education represents maximal 1 year of completed vocational education;**
>
> **[c] Vocational education represents more than 1 year of completed vocational education without completed academic education;**
>
> **[d] Academic education represents a bachelor's degree from a university or university of applied sciences.**

R.1.11. Table 3
Does those NN in the 'Composite job demands' column refer to zero? Please provide the numbers the authors can obtain from the analysis results.

NN in the bottleneck table refers to 'not necessary'. A clarifying footnote has been added:

> *** NN: not necessary, meaning that for a level of 1 (never) on work-related emotional exhaustion (Y) no minimum level of psychosocial work adversity (X) is required.**

Both reviewers had comments on this table and therefore it has been compressed, the complete bottleneck table with 0.2 substeps can be found in the supplementary information.

R.1.12. Effect size
I suggest that all the effect size (ES) reported in this section should be followed by their 95% CI of relevant ES.

Since NCA is essentially not a statistical but a mathematical method, no confidence interval for NCA exists. Uncertainties are not expressed in probabilities but in 'cardinalities' (e.g., number of cases that do not comply: exceptions).

However, the p-value of NCA permutation tests and the 'ceiling accuracy' can provide indications about the credibility of the (mathematically) estimated ceiling line. Both were very stable in our case.

The above mentioned explanation of the ‘lack of’ a confidence interval is added to the manuscript in lines 193-195.

R.1.13 Discussion
This section should have a limitation paragraph.
The limitations have been described in the methodological considerations paragraph:

> **Methodological considerations**
> **A limitation of our study is that it is based on cross-sectional data. The occurrence of reverse and reciprocal causality between mental health outcomes and psychosocial adversities is still a matter of debate 19, 20 and work-related emotional exhaustion has also been shown to change perceptions of job demands and resources[21]. Consequently, we cannot rule out that high emotional exhaustion itself contributes to higher (experienced) psychosocial work adversities. In general, necessary conditions that are identified in a cross-sectional study may be causally interpreted only if there is theoretical support for it and if reverse causality is considered implausible. The credibility of a causal necessity relationship increases with a time-lagged, longitudinal or experimental study design[22]22. Further research of this type is needed to rule out reciprocal or reversed causality.**
>
> **References**

> **21. Guthier C, Dormann C and Voelkle MC. Reciprocal effects between job stressors and burnout: A continuous time meta-analysis of longitudinal studies. Psychol Bull 2020; 146: 1146-1173. 20201029. DOI: 10.1037/bul0000304.**
> **22. Dul J. Advances in necessary condition analysis. Downloaded on Jan 2021; 21: 2022.**

R.1.14 Ethics approval
In lines 380-382, please add the approval number.

This was added, so that the paragraph now reads:

> Ethics approval
> **The Netherlands Working Conditions Survey from which this study draws was not subject to the requirements of the Dutch Medical Research Involving Human Subjects Act as declared by the Internal Review Board of the Netherlands Organisation for Applied Scientific Research TNO (approval number 2018-066).**

Reviewer: 2
Dr. Gergely Feher, University of Pécs

Comments to the Author:
This manuscript offers an innovative and valuable contribution to occupational health research by applying Necessary Condition Analysis (NCA) to assess the role of psychosocial work adversities in emotional exhaustion among young workers. The paper is theoretically well-grounded, methodologically rigorous, and highly relevant to both academic and policy audiences. I commend the authors for the clarity of writing, the robustness of their findings, and the thoughtful interpretation of results.

Major Strengths
Novel Methodological Approach:

The use of NCA introduces a fresh analytical lens to occupational mental health research. By focusing on necessary (rather than probabilistic) conditions, the study adds a deterministic perspective to the literature, which has largely been dominated by average-effect models such as regression or SEM. The authors also correctly emphasize the practical utility of NCA in formulating policy thresholds.

Robustness of Results:
The main findings are replicated across seven waves of nationally representative survey data (2014–2020), thereby greatly enhancing the external validity and credibility of the conclusions.

Theoretical Integration:
The manuscript is well anchored in the Job Demands–Resources (JDR) model, which is appropriately adapted to the context of necessity logic. The concept of equifinality is correctly invoked to justify composite score usage.

Clear Policy Implications:
The findings suggest that without psychosocial adversity, high levels of emotional exhaustion are virtually absent. This is a powerful message for occupational health policy and workplace design.

We would like to thank the reviewer for the time, attention and effort devoted to reviewing our work.

Major Concerns

R.2.1 Cross-sectional Design and Causality:
Although the authors acknowledge this limitation, it is worth reiterating that the cross-sectional nature of the study limits causal inference, even under necessity logic. It is important to explicitly state that NCA can detect statistical necessity, but not causal necessity without temporal precedence.

Thank you for pointing this out, we have added this point to lines 334-335 in the discussion, as follows:

> **As a test for robustness, we replicated the findings in seven earlier waves of the same survey. All results were statistically significant and effect sizes were small to medium (see Supplementary Information). Despite the observed necessity, to infer causal necessity beyond theoretical reasoning a longitudinal dataset with multiple datapoints per participant would be helpful.**

R.2.2. Operationalization of High vs. Low Resources:
The median split used to dichotomize job resources (for hypothesis 2) may reduce statistical power and introduce arbitrary classification. Sensitivity analyses using alternative cut-offs (e.g., tertiles or quartiles) could enhance the robustness of the subgroup comparisons.

We agree with the reviewer that this could be a risk and we have conducted two additional sensitivity analyses with quartiles and 20n tiles.

In the sensitivity analysis using job resources based on quartiles (figure 1), we see that the higher the resource score the larger the effect size. In the 20n tiles (figure 2), the trend is the

same, but for some 20n tiles there are outliers that lower the empty-space/effect size. The comparison hence seems to be robust.

Since the quartile figure is most informative, we added the quartile sensitivity analysis to the supplementary information and refer to this in the article, as follows:

> Lastly, we performed an additional sensitivity analysis using quartiles as a cut-off instead of median split for creating groups of young workers with lower versus higher levels of job resources. We could replicate our finding, and found in addition that the higher the job resource score, the larger the effect size (Supplementary file, Figure 1; q1 = .10; q2=.12; q3=.15; q4=.20; all p-values <.001).

Figure 1.

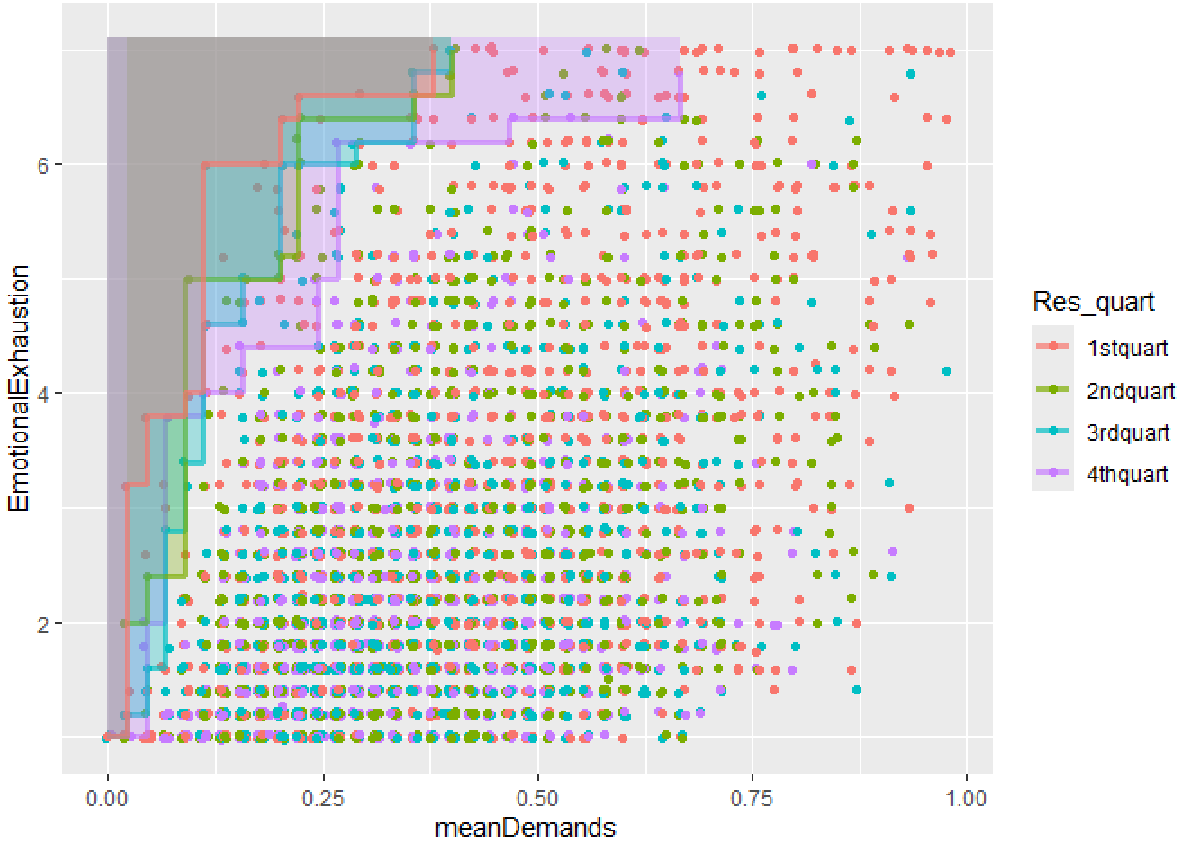

effect size ce_fdh	p-value	quartile
0.101	0.001	1stq
0.124	0.001	2ndq
0.154	0.001	3rdq
0.199	0.001	4thq

Figure 2.

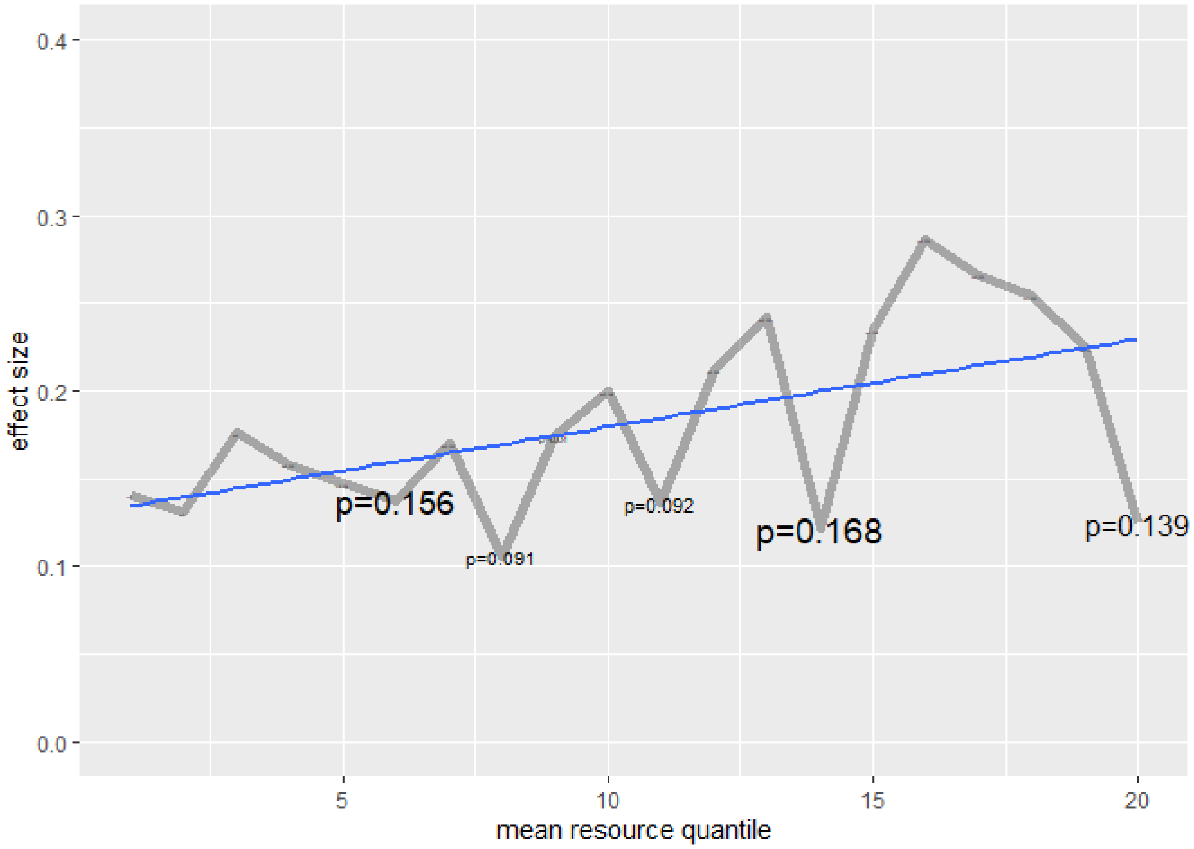

R.2.3 Interpretability of Composite Scores:
While the composite indices are theoretically justified, the practical interpretation of what constitutes a “high” or “low” adversity score (e.g., 0.15) may be ambiguous for readers or policymakers. The discussion would benefit from more concrete examples or thresholds.

We agree with the reviewer that it is notoriously difficult to interpret scores of psychosocial work adversities in terms of risk-thresholds for mental health. We have added this notion and more context to this long standing scientific debate on the topic. We have also added a proposed way forward to get to an agreement on high and low adversity scores, based on a recent discussion paper published in SJWEH (see lines 303-312, marked text is new):

> **Moreover, the levels of x and y are shown in the bottleneck table, but the interpretation of what level of exposure and outcome is (too) high needs discussion among scholars and practitioners as well as between managers and employees within organizations: is it acceptable to experience work-related emotional exhaustion several times a year, monthly or every week, and under what conditions? An interdisciplinary group of authors from science, practice and policy described in a recent discussion paper {Pauli, #35} the well-known difficulty of determining occupational exposure limits for psychosocial risk factors in the workplace. The authors proposed a stepwise process of joint action with all relevant stakeholders (e.g., scientists, practitioners, policy-makers), ranging from *conceptual agreement* to experimental and epidemiological evidence, leading to *consensus* and ultimately the establishment of *occupational exposure limits*. Our results, as well as future NCA analyses for other subgroups or even the entire working population, could inform this process and**

complement the proposed NOAEL (no-observed-adverse-effect level) and LOAEL (lowest-observed-adverse-effect level) methods, as NCA results apply to (virtually) all cases.

Pauli R, Lang J, Müller A, Taibi Y, Kraus T, Metzler Y. Requirements for occupational exposure limits in psychosocial risk assessment: What we know, what we don't know and what we can learn from other disciplines. Scandinavian Journal of Work, Environment & Health. 2025 Sep 4.

Minor Points and Recommendations
R.2.4 Please clarify in the Methods whether Cronbach's alphas were calculated on the study sample or obtained from prior validation studies.
Cronbach's alphas were calculated based on our own sample. This is added to the manuscript in the following manners:

- **In the paragraph on the outcome variable: work-related emotional exhaustion: Internal consistency of the scale in this sample was very good (Cronbach's alpha = .89).**
- **In the paragraph on exposure variables - job demands and job resources variables: Cronbach's alphas for all scales were calculated to establish the internal reliability, with satisfactory to good results (lowest Cronbach's alpha=.75; highest Cronbach's alpha = .85).**
- **As a note to table 1, which has been moved to the supplementary information as suggested by another reviewer: Cronbach's alphas are calculated for the subsample used in this study.**

R.2.5 The bottleneck tables are informative, but their layout could be improved for readability (e.g., alignment and value formatting).

The layout of the bottleneck table is simplified, see table 2. The former table has been moved to the supplementary information for completeness, and the layout has been improved for readability.

R.2.6 The rationale for using the CE-FDH ceiling line could be briefly elaborated with references or figures for readers unfamiliar with the method.
We briefly described the rationale in the method section, lines 185-186, and we've added a reference to the advances in NCA book by prof. dr. Jan Dul for the interested reader:

We selected the stepwise CE-FDH ceiling line because the border was not linear [21].

R.2.7 Consider including a figure summarizing the conceptual model (e.g., hypothesized necessity logic within the JDR framework).

Thank you for this suggestion, we have added the following figure to the manuscript depicting the necessity hypotheses within the JDR framework.

Figure 1. Necessity hypotheses within the Job Demands Resources framework

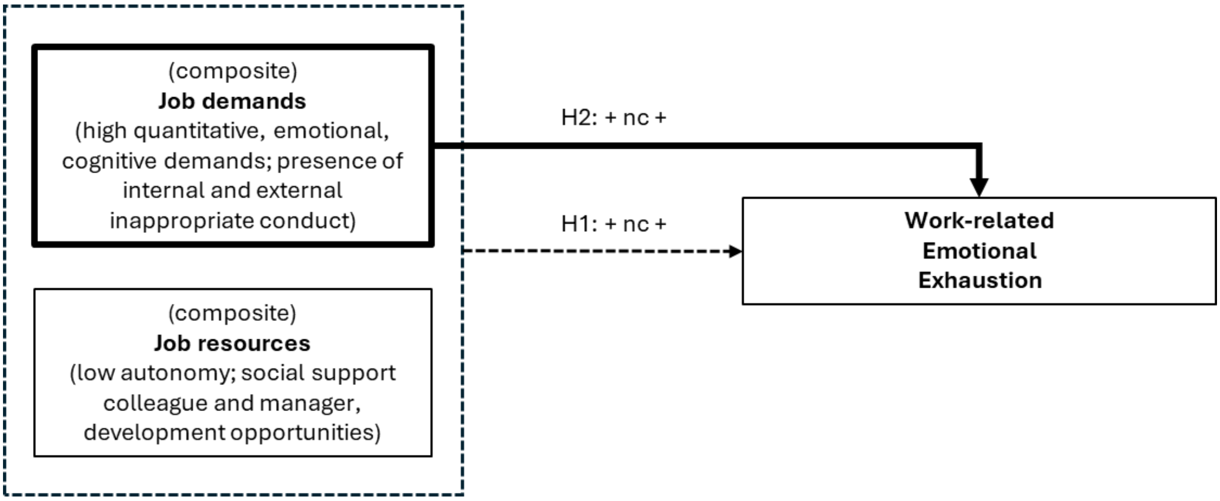

VERSION 2 - REVIEW

**Reviewer**	**2**
**Name**	**Feher, Gergely**
**Affiliation**	**University of Pécs, Centre for Occupational**
**Date**	**15-Oct-2025**
**COI**	

The authors have addressed the raised concerns, and the manuscript now meets the standards for publication.
